# Supplementary material for: A Sparse and Low-Rank Regression Model for Identifying the Relationships Between DNA Methylation and Gene Expression Levels in Gastric Cancer and the Prediction of Prognosis
Source: Genes (Basel). 2021 Jun 2;12(6):854. doi: 10.3390/genes12060854 (PMC8228406; doi:10.3390/genes12060854)
Supplement: Supplementary file 1 [file genes-12-00854-s001.zip › Supplementary/Figure S1-S29.pdf]

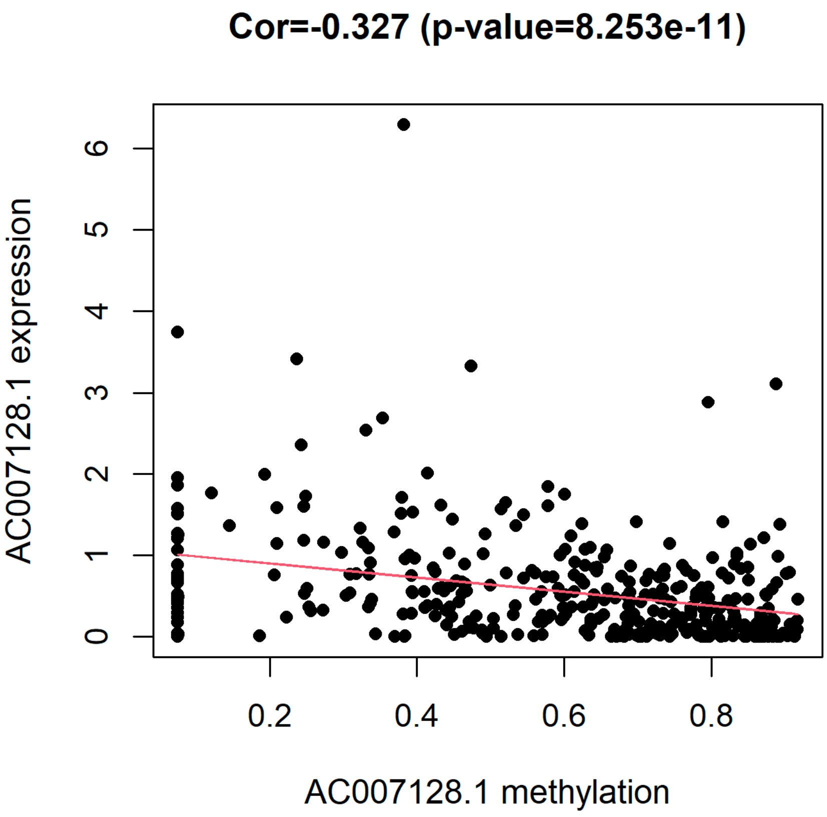
Cor=-0.327 (p-value=8.253e-11)
AC007128.1 expression
0
1
2
3
4
5
6
0.2
0.4
0.6
0.8
AC007128.1 methylation

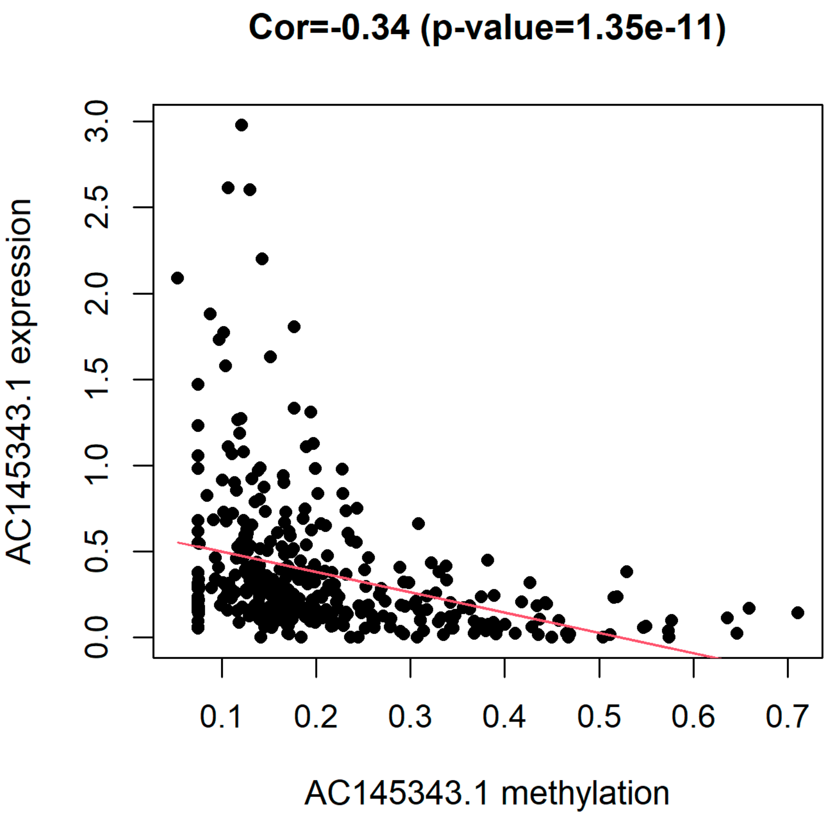
Cor=-0.34 (p-value=1.35e-11)
AC145343.1 expression
0.0
0.5
1.0
1.5
2.0
2.5
3.0
0.1
0.2
0.3
0.4
0.5
0.6
0.7
AC145343.1 methylation

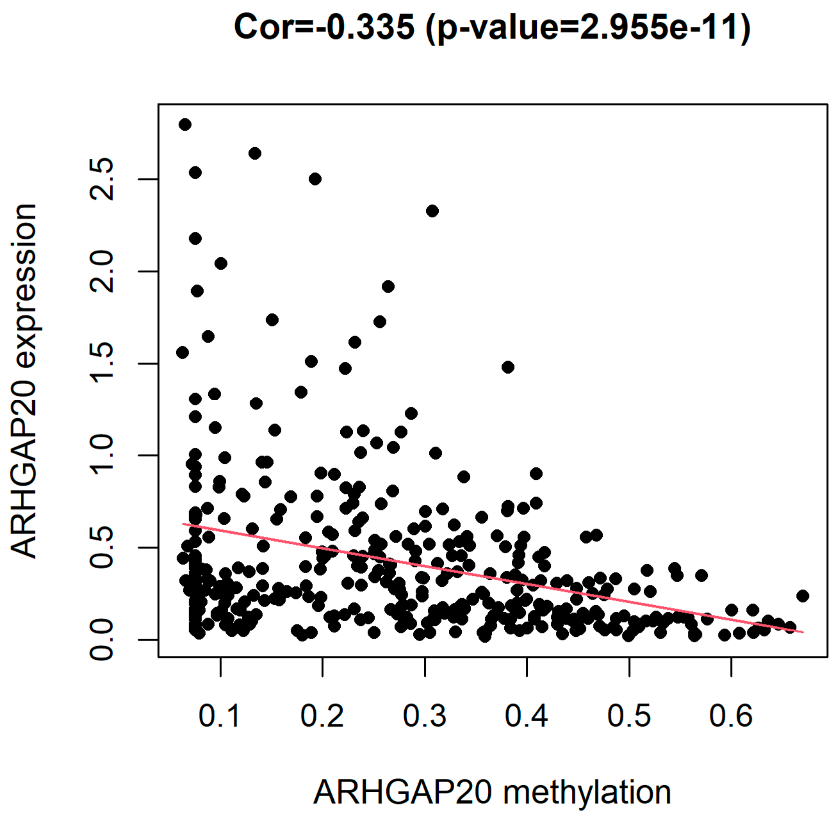
Cor=-0.335 (p-value=2.955e-11)
ARHGAP20 expression
0.0
0.5
1.0
1.5
2.0
2.5
0.1
0.2
0.3
0.4
0.5
0.6
ARHGAP20 methylation

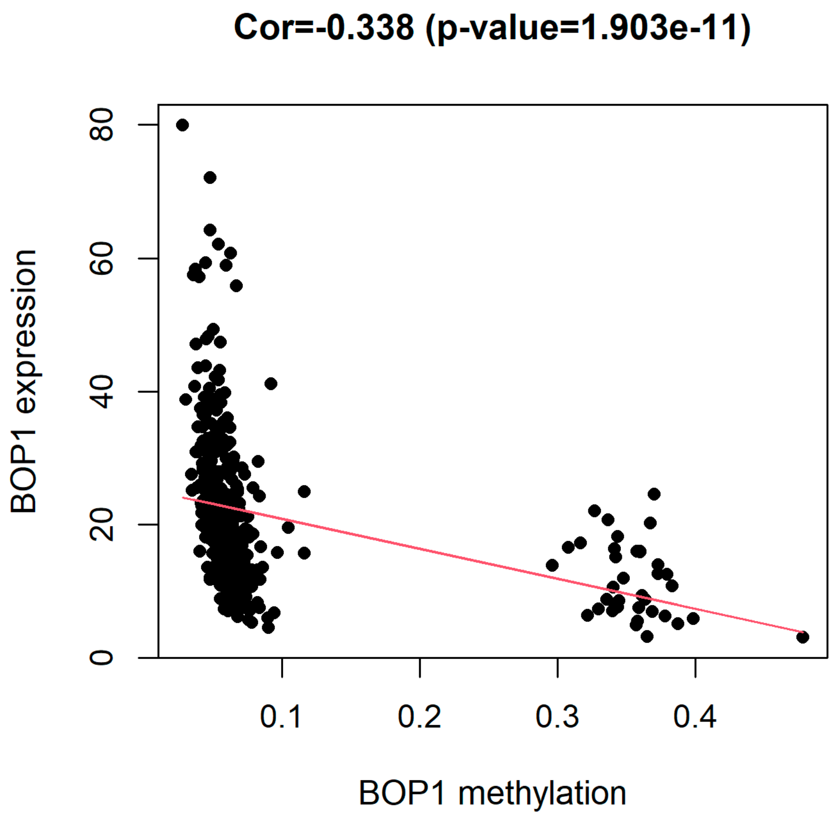
Cor=-0.338 (p-value=1.903e-11)
BOP1 expression
0
20
40
60
80
0.1
0.2
0.3
0.4
BOP1 methylation

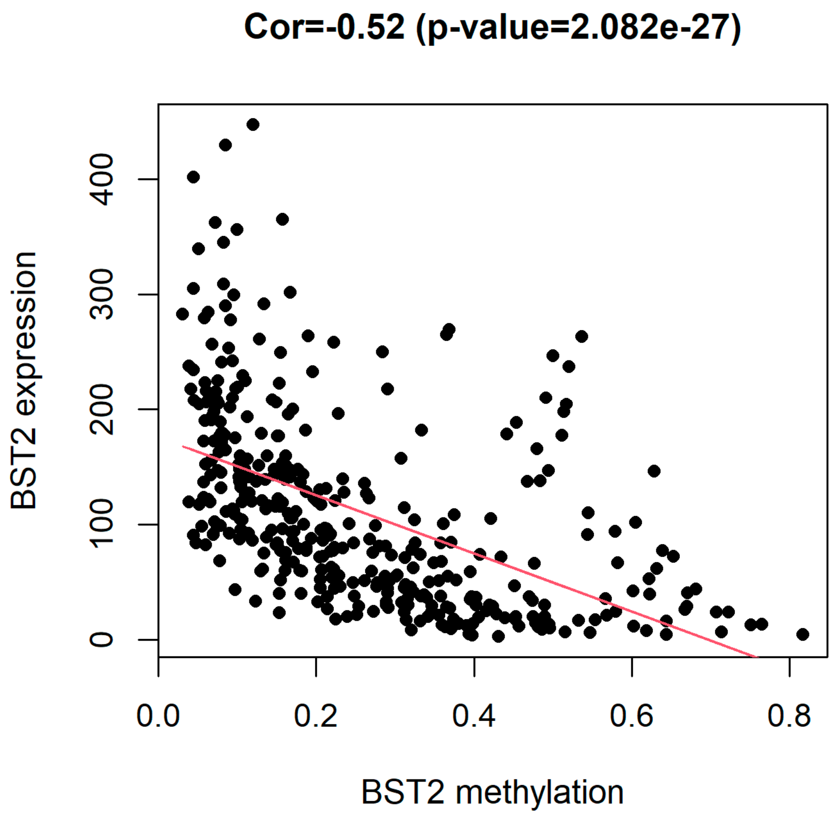
Cor=-0.52 (p-value=2.082e-27)
BST2 expression
BST2 methylation
0
100
200
300
400
0.0
0.2
0.4
0.6
0.8

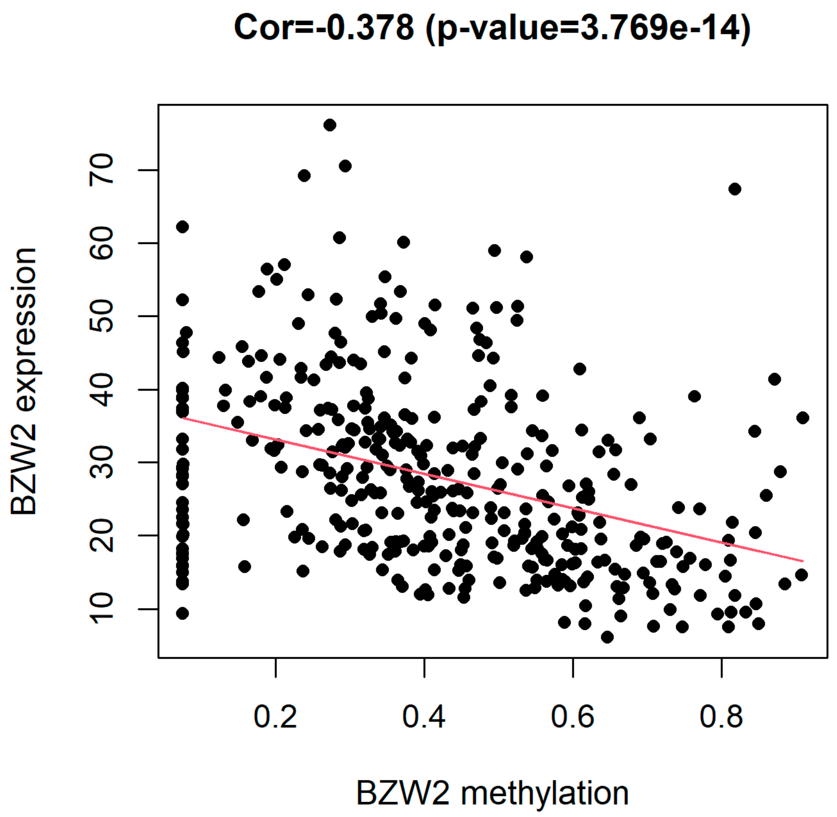
Cor=-0.378 (p-value=3.769e-14)
BZW2 expression
BZW2 methylation
10
20
30
40
50
60
70
0.2
0.4
0.6
0.8

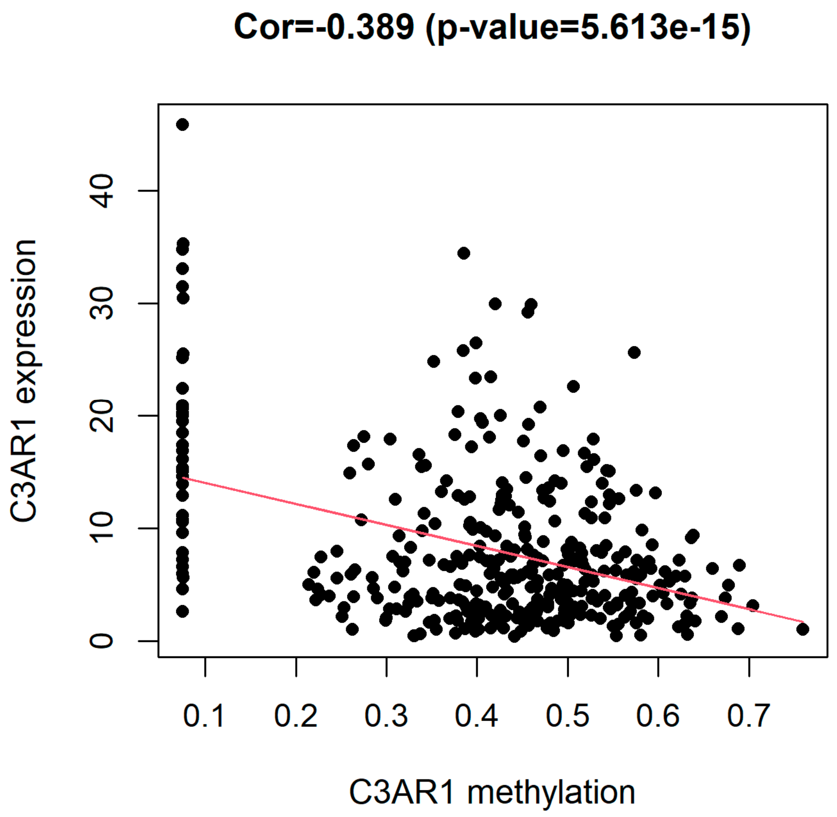
Cor=-0.389 (p-value=5.613e-15)
C3AR1 expression
0
10
20
30
40
0.1
0.2
0.3
0.4
0.5
0.6
0.7
C3AR1 methylation

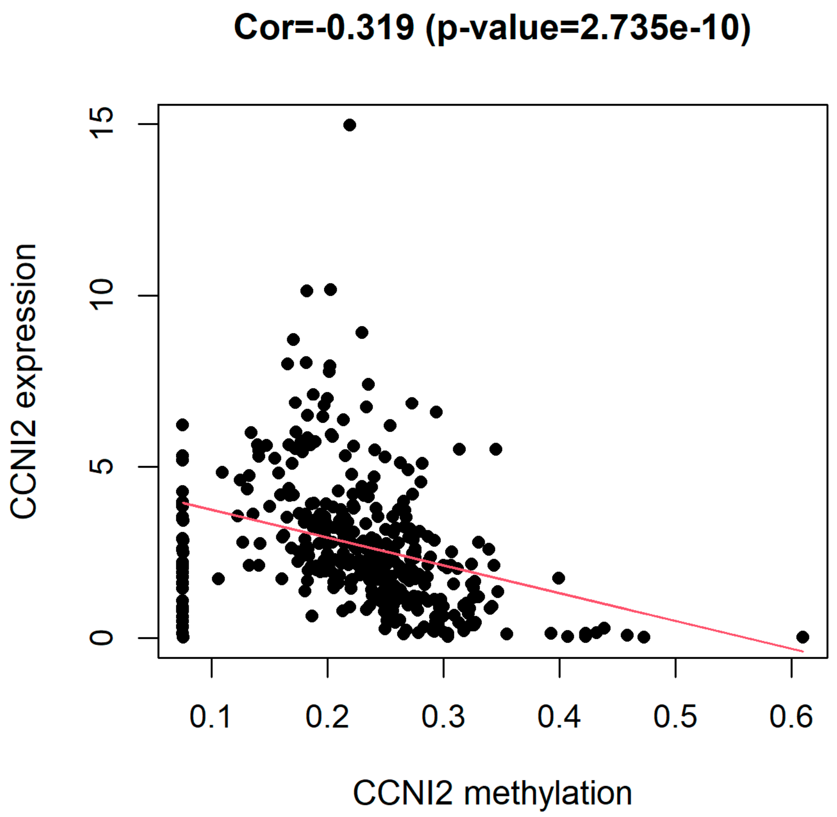
Cor=-0.319 (p-value=2.735e-10)
CCNI2 expression
0
5
10
15
0.1
0.2
0.3
0.4
0.5
0.6
CCNI2 methylation

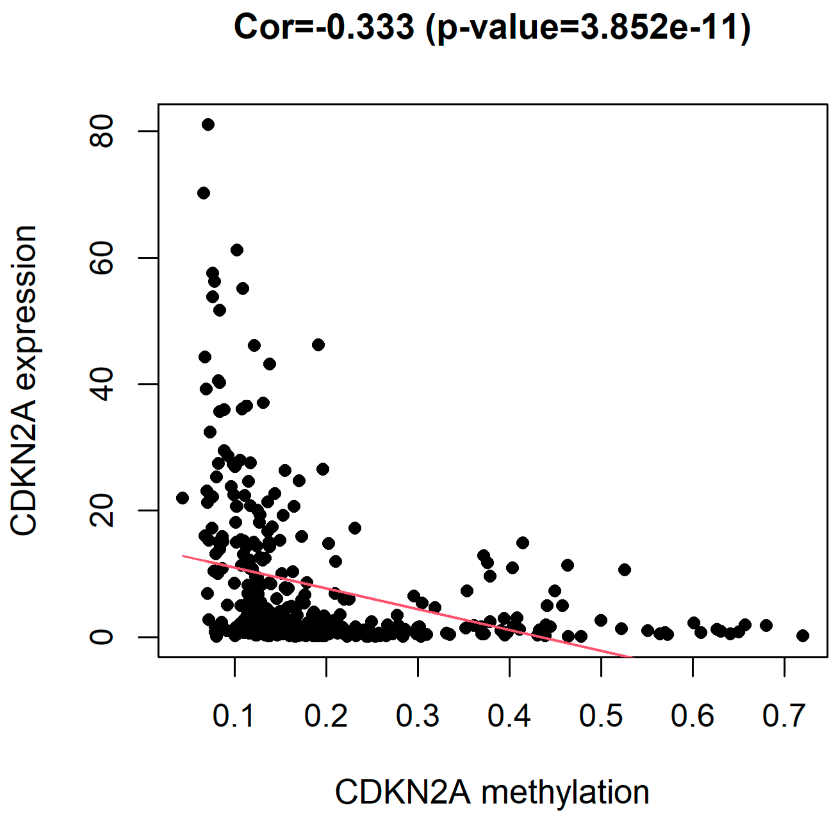

Cor=-0.333 (p-value=3.852e-11)
CDKN2A expression
0
20
40
60
80
0.1
0.2
0.3
0.4
0.5
0.6
0.7
CDKN2A methylation

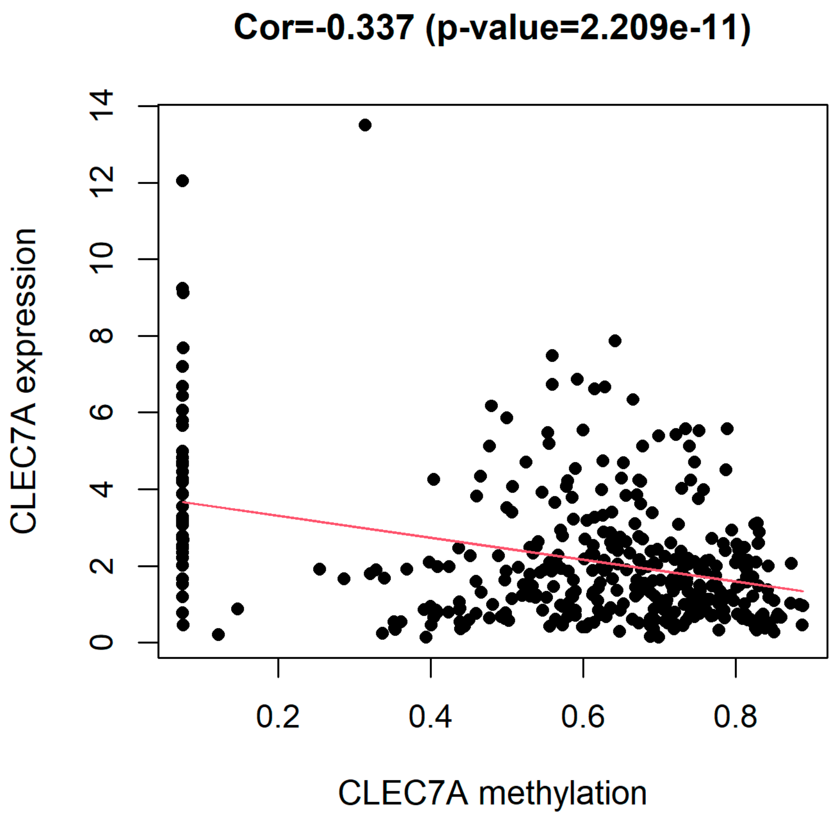

Cor=-0.337 (p-value=2.209e-11)
CLEC7A expression
0
2
4
6
8
10
12
14
0.2
0.4
0.6
0.8
CLEC7A methylation

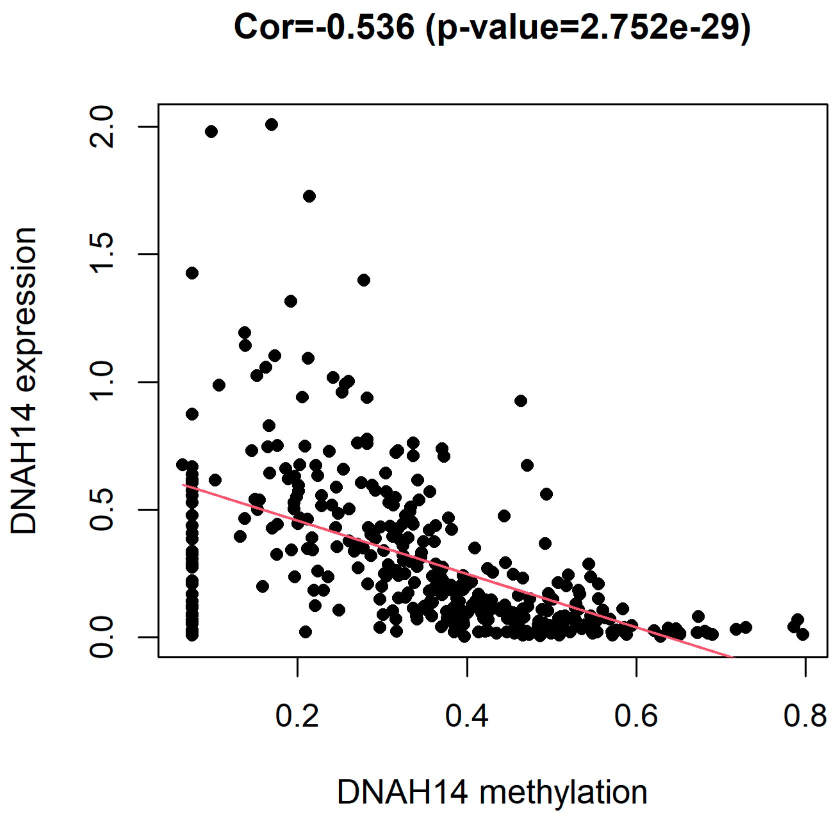

Cor=-0.536 (p-value=2.752e-29)
DNAH14 expression
0.0
0.5
1.0
1.5
2.0
0.2
0.4
0.6
0.8
DNAH14 methylation

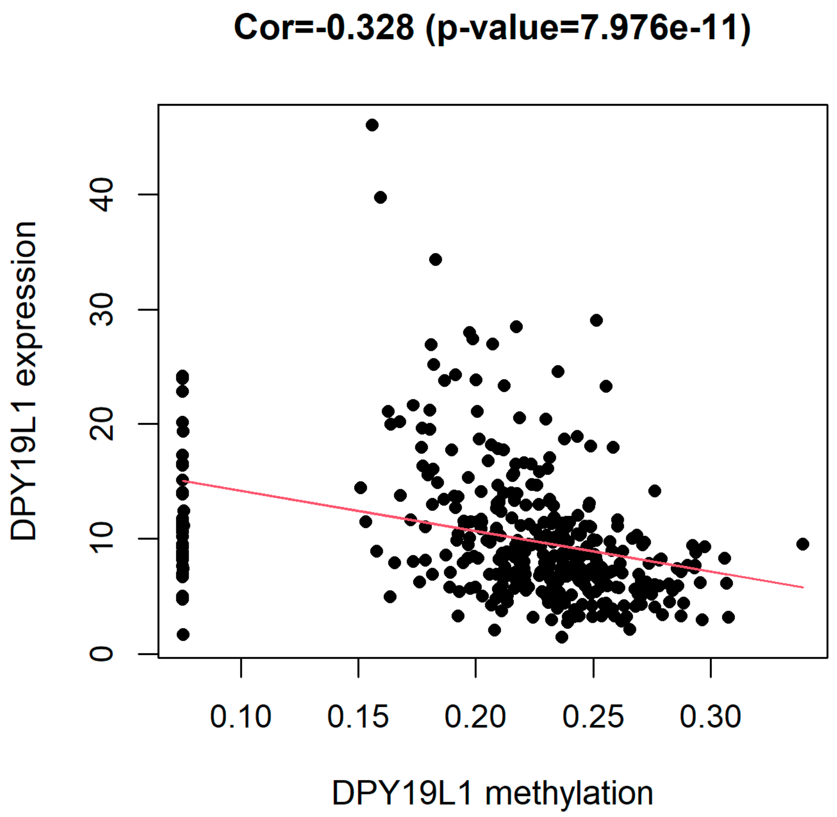

Cor=-0.328 (p-value=7.976e-11)
DPY19L1 expression
0
10
20
30
40
0.10
0.15
0.20
0.25
0.30
DPY19L1 methylation

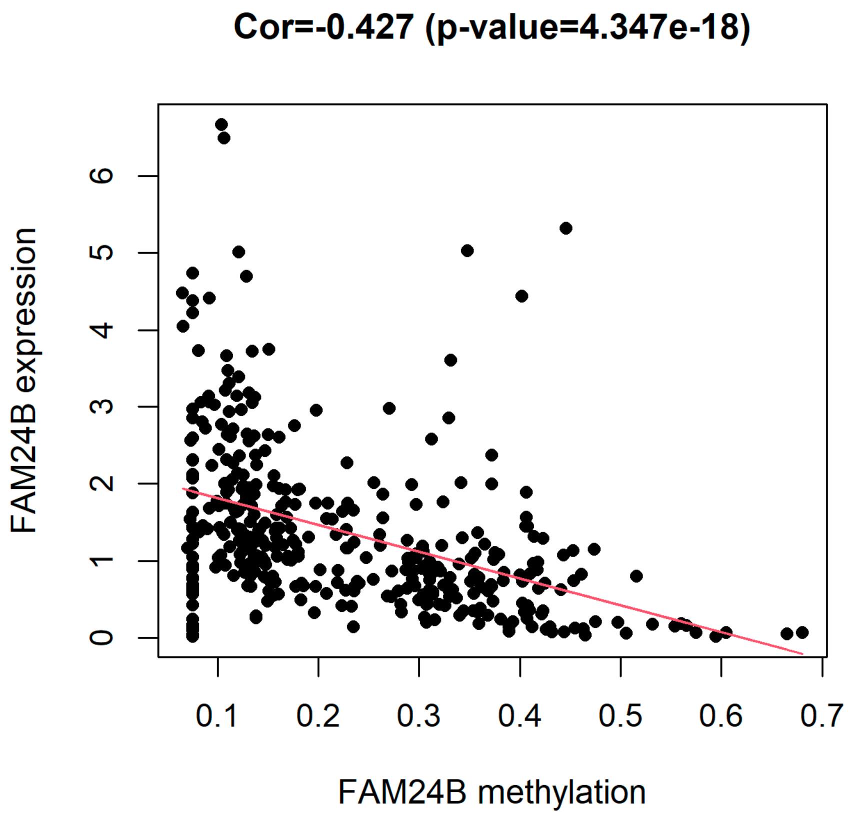

Cor=-0.427 (p-value=4.347e-18)
FAM24B expression
FAM24B methylation
0.1
0.2
0.3
0.4
0.5
0.6
0.7
0
1
2
3
4
5
6

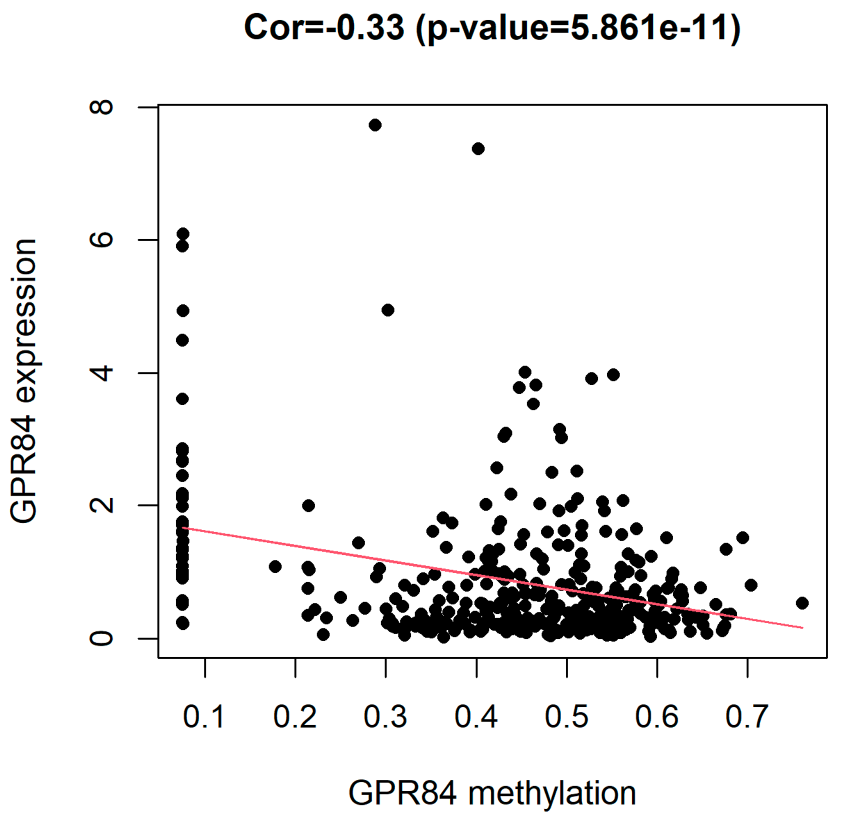

Cor=-0.33 (p-value=5.861e-11)
GPR84 expression
GPR84 methylation
0.1
0.2
0.3
0.4
0.5
0.6
0.7
0
2
4
6
8

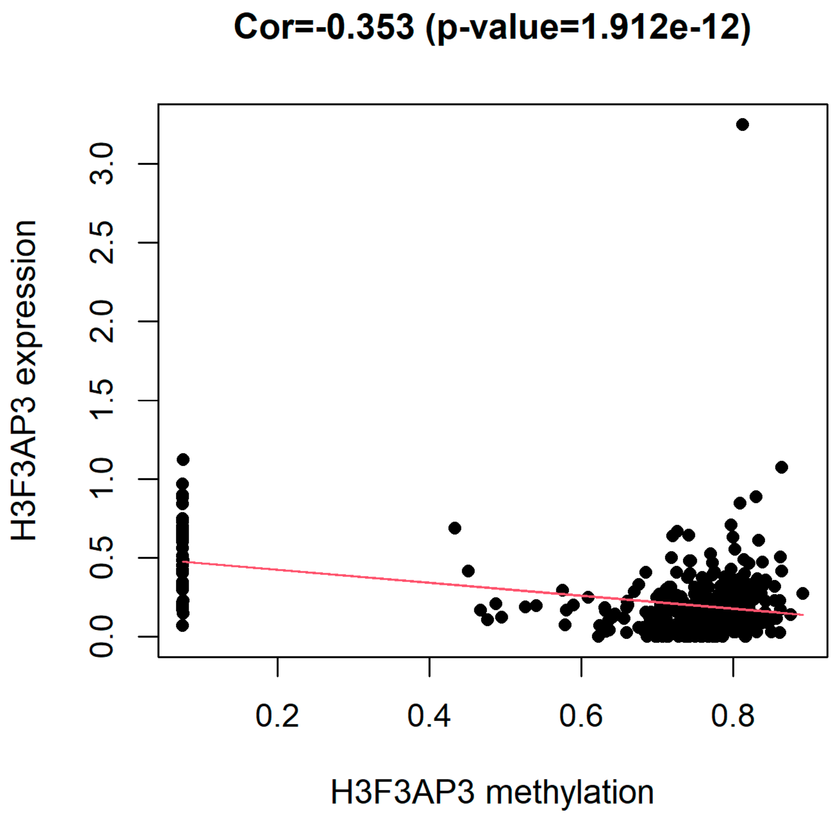

Cor=-0.353 (p-value=1.912e-12)
H3F3AP3 expression
0.0
0.5
1.0
1.5
2.0
2.5
3.0
0.2
0.4
0.6
0.8
H3F3AP3 methylation

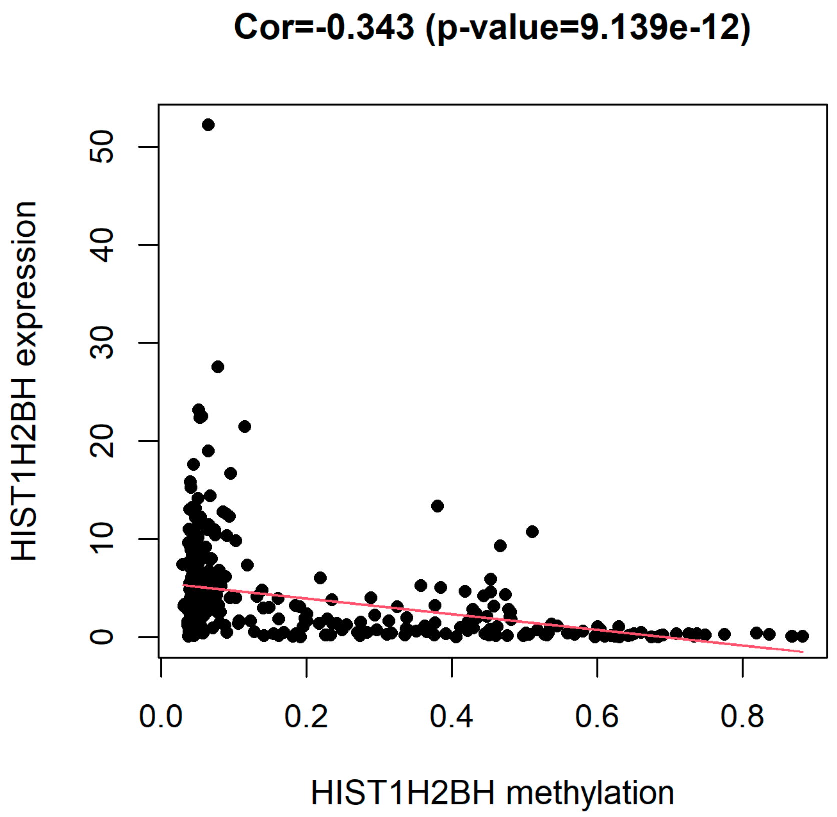

Cor=-0.343 (p-value=9.139e-12)
HIST1H2BH expression
0
10
20
30
40
50
0.0
0.2
0.4
0.6
0.8
HIST1H2BH methylation

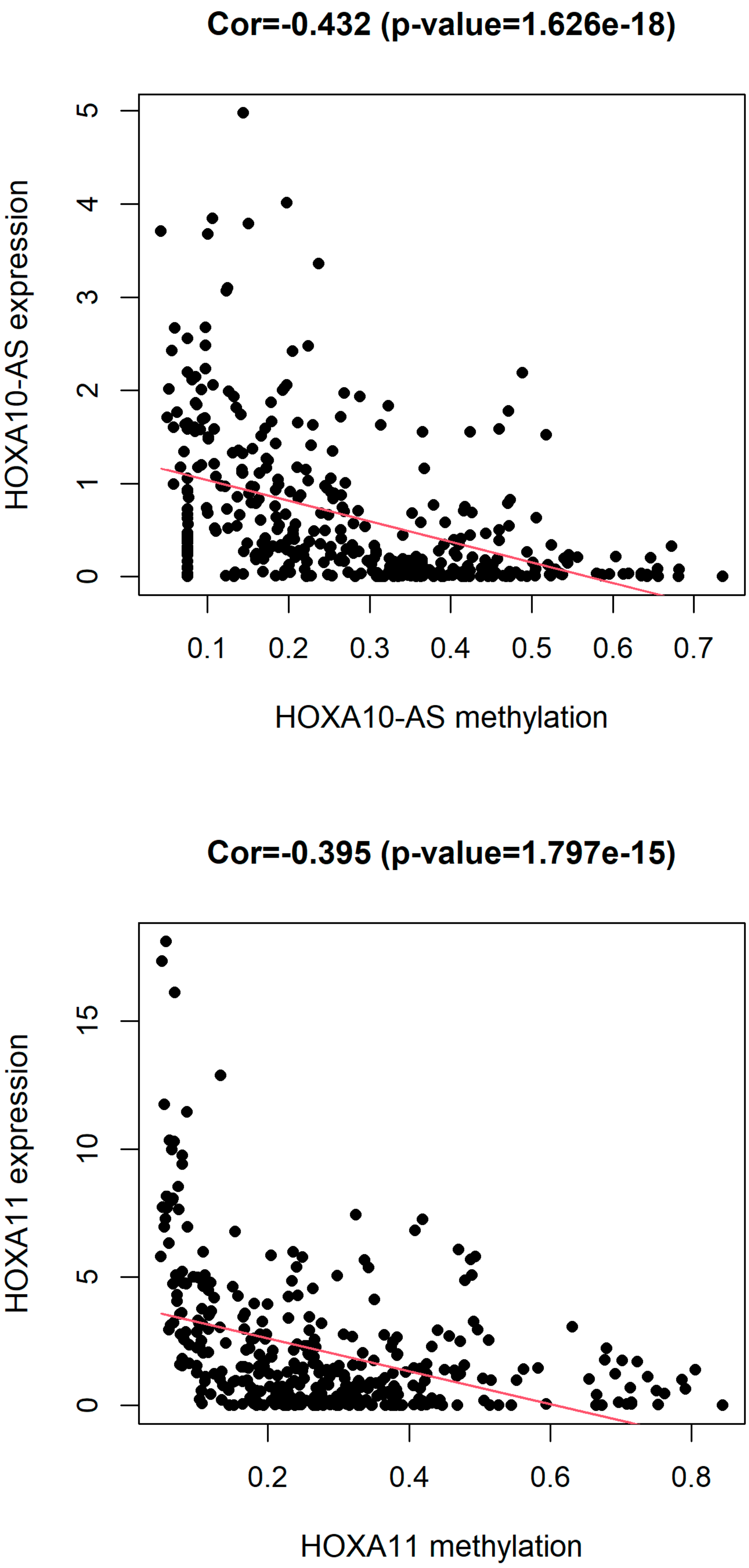
Cor=-0.432 (p-value=1.626e-18)
HOXA10-AS expression
0
1
2
3
4
5
0.1
0.2
0.3
0.4
0.5
0.6
0.7
HOXA10-AS methylation
Cor=-0.395 (p-value=1.797e-15)
HOXA11 expression
0
5
10
15
0.2
0.4
0.6
0.8
HOXA11 methylation

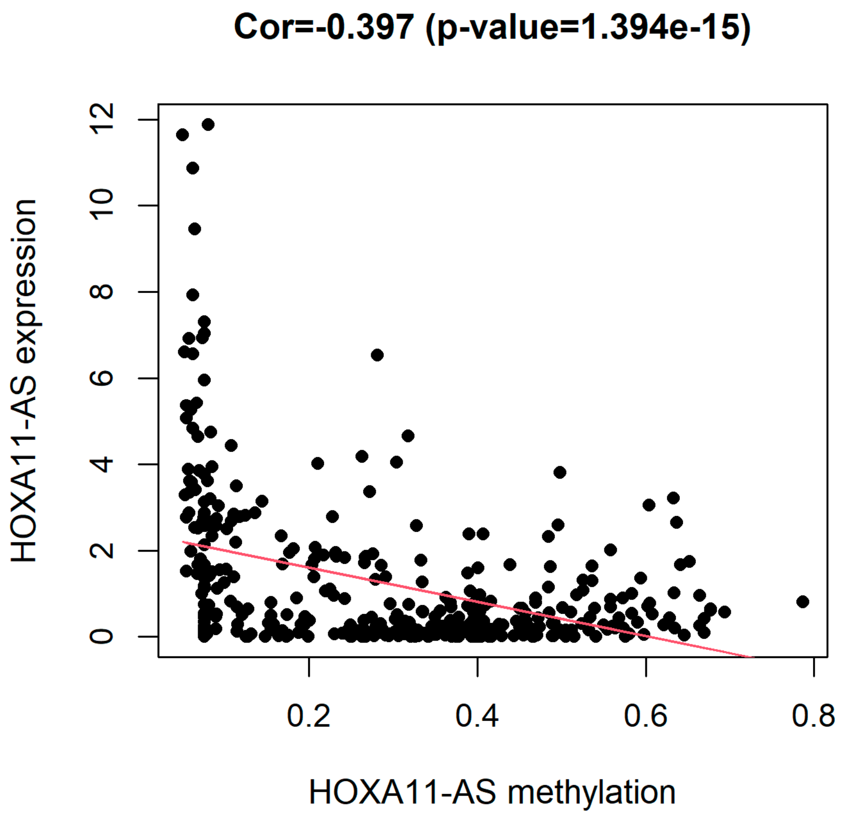
Cor=-0.397 (p-value=1.394e-15)
HOXA11-AS expression
0
2
4
6
8
10
12
0.2
0.4
0.6
0.8
HOXA11-AS methylation

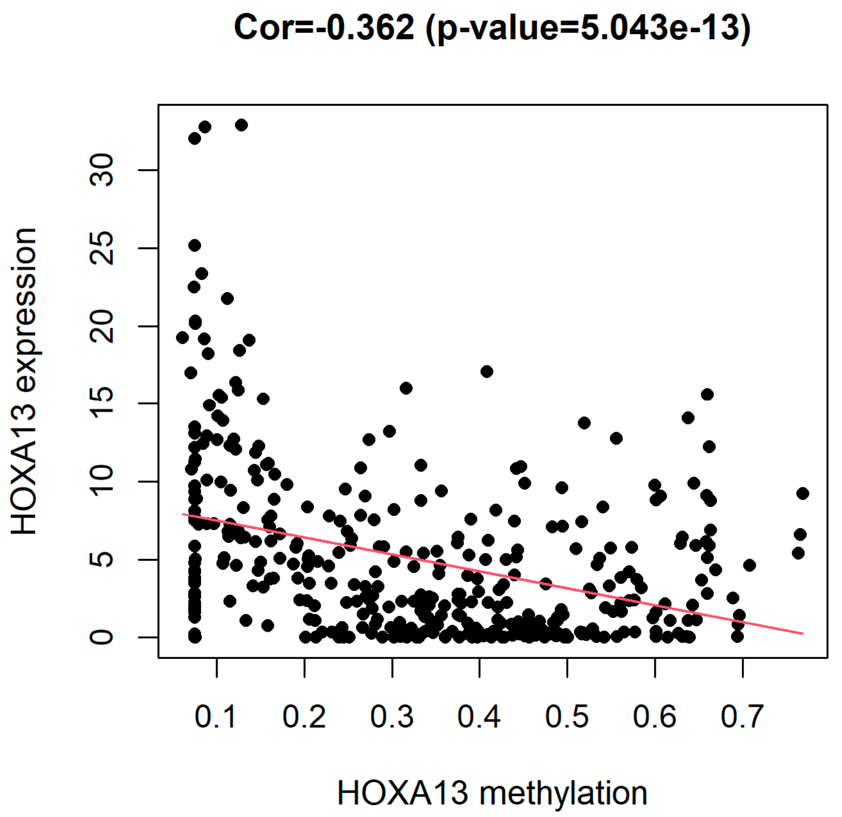
Cor=-0.362 (p-value=5.043e-13)
HOXA13 expression
0
5
10
15
20
25
30
0.1
0.2
0.3
0.4
0.5
0.6
0.7
HOXA13 methylation

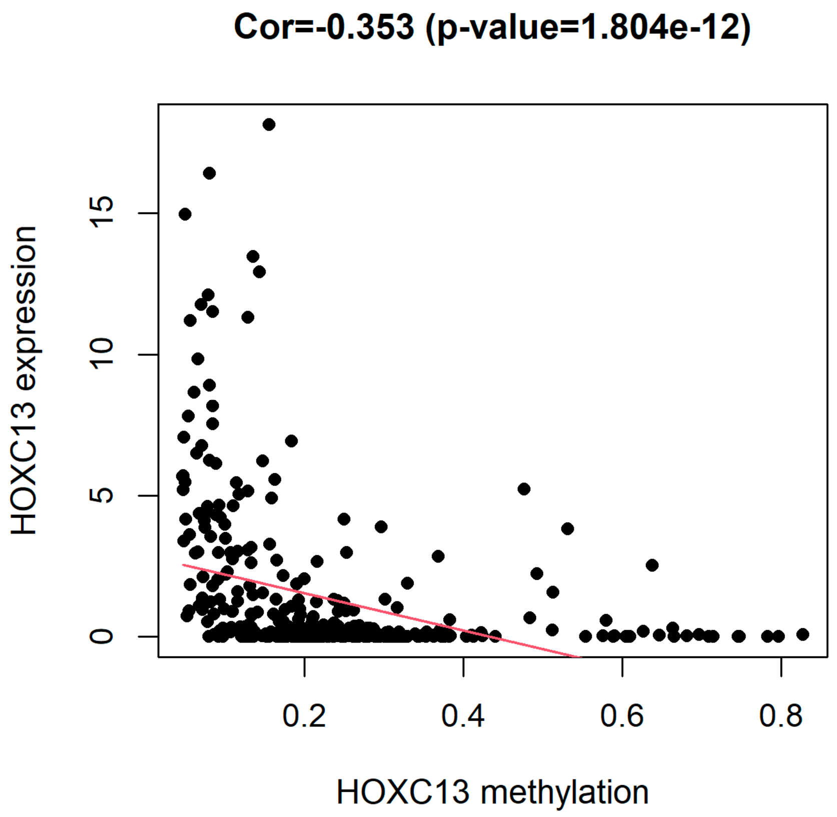

Cor=-0.353 (p-value=1.804e-12)
HOXC13 expression
0
5
10
15
0.2
0.4
0.6
0.8
HOXC13 methylation

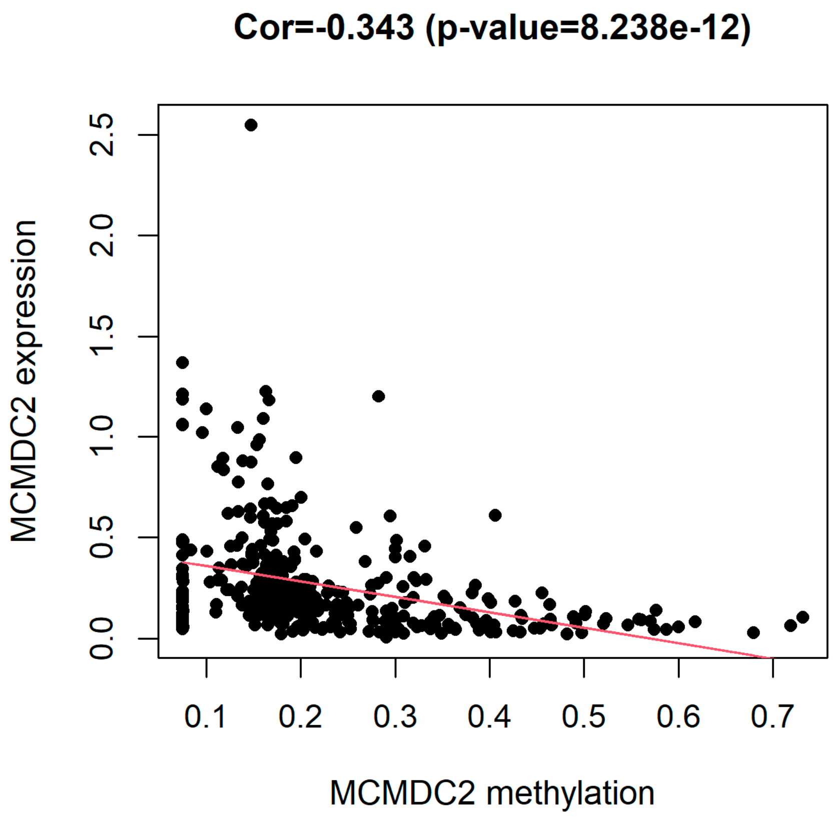

Cor=-0.343 (p-value=8.238e-12)
MCMDC2 expression
0.0
0.5
1.0
1.5
2.0
2.5
0.1
0.2
0.3
0.4
0.5
0.6
0.7
MCMDC2 methylation

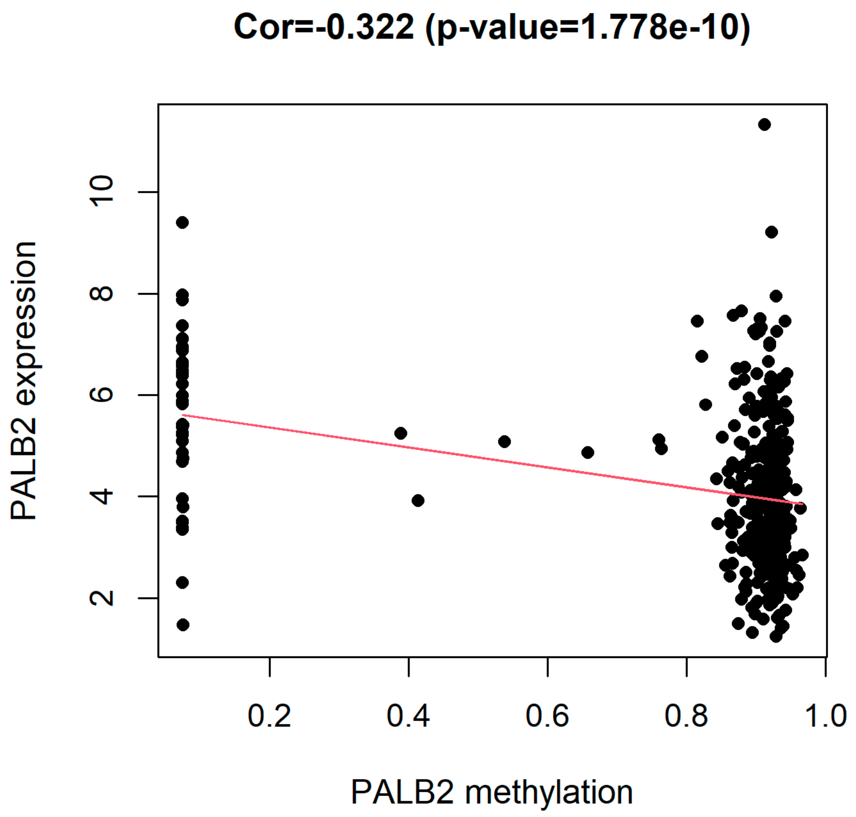
Cor=-0.322 (p-value=1.778e-10)
PALB2 expression
PALB2 methylation
2
4
6
8
10
0.2
0.4
0.6
0.8
1.0

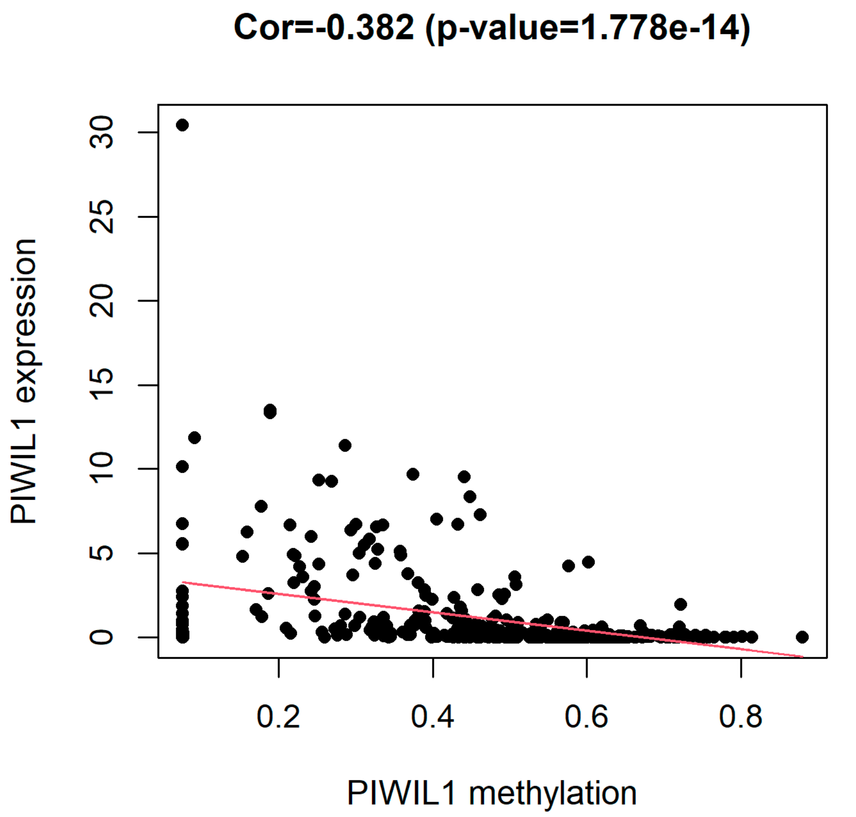
Cor=-0.382 (p-value=1.778e-14)
PIWIL1 expression
PIWIL1 methylation
0
5
10
15
20
25
30
0.2
0.4
0.6
0.8

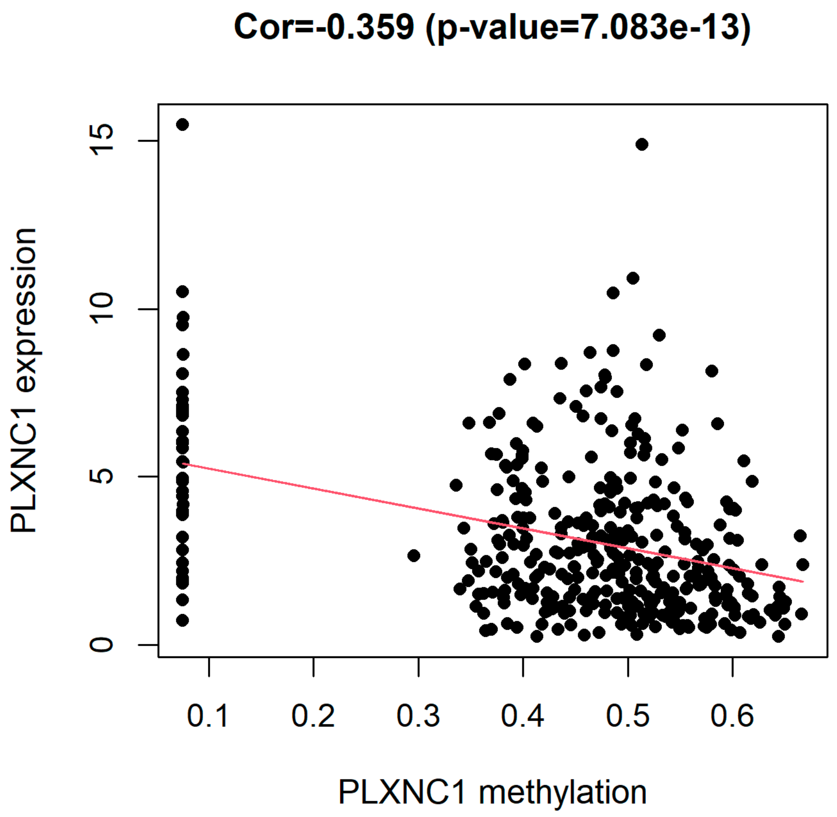

Cor=-0.359 (p-value=7.083e-13)
PLXNC1 expression
0
5
10
15
0.1
0.2
0.3
0.4
0.5
0.6
PLXNC1 methylation

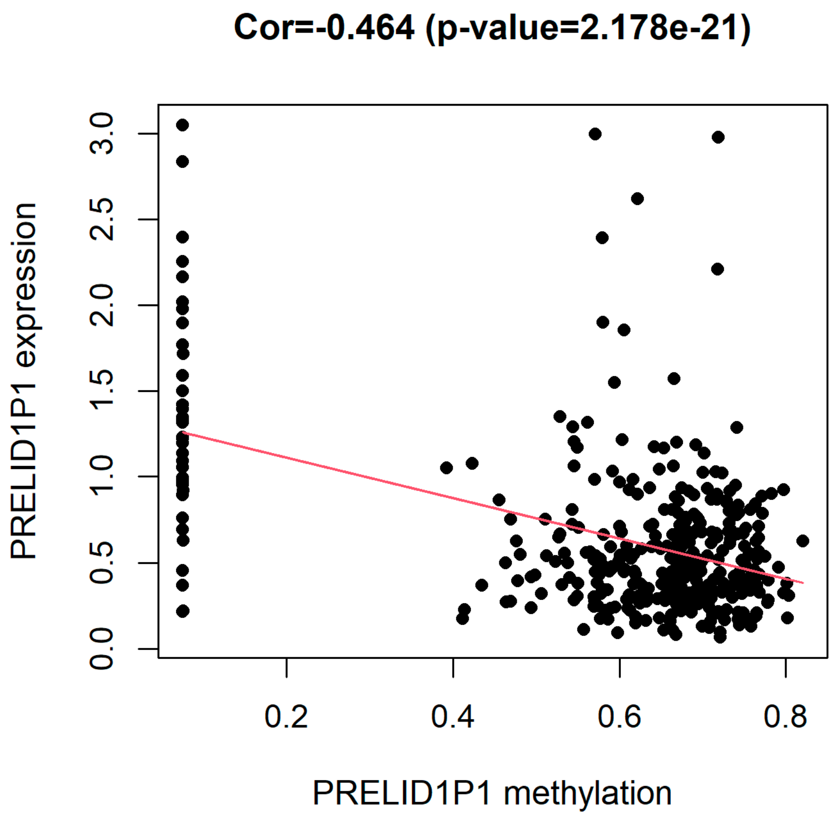

Cor=-0.464 (p-value=2.178e-21)
PRELID1P1 expression
0.0
0.5
1.0
1.5
2.0
2.5
3.0
0.2
0.4
0.6
0.8
PRELID1P1 methylation

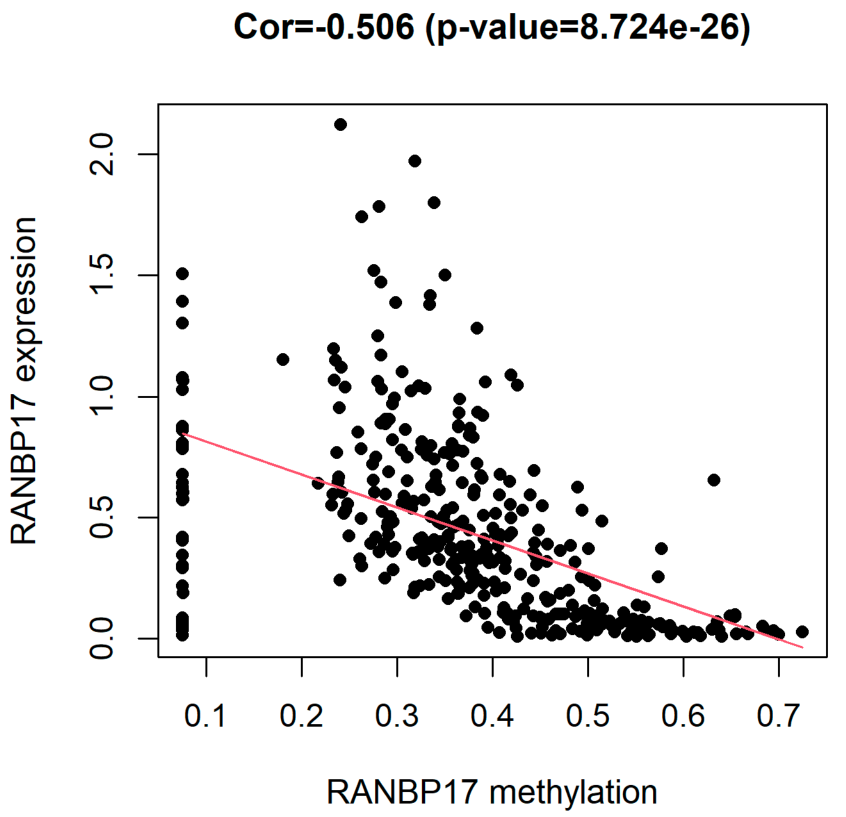

Cor=-0.506 (p-value=8.724e-26)
RANBP17 expression
0.0
0.5
1.0
1.5
2.0
0.1
0.2
0.3
0.4
0.5
0.6
0.7
RANBP17 methylation

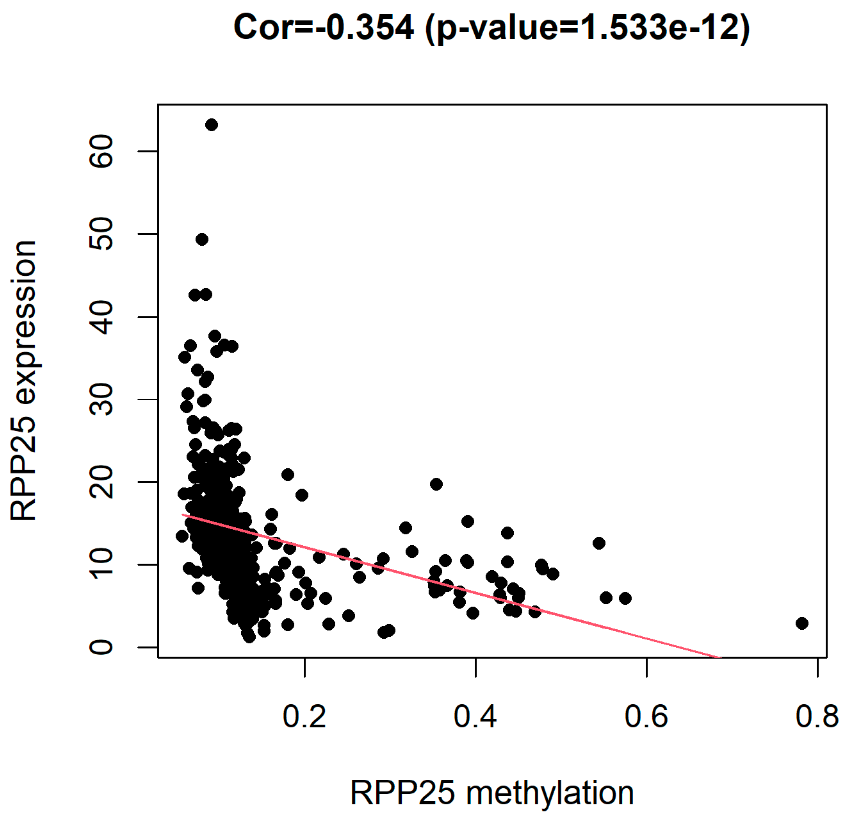

Cor=-0.354 (p-value=1.533e-12)
RPP25 expression
0
10
20
30
40
50
60
0.2
0.4
0.6
0.8
RPP25 methylation

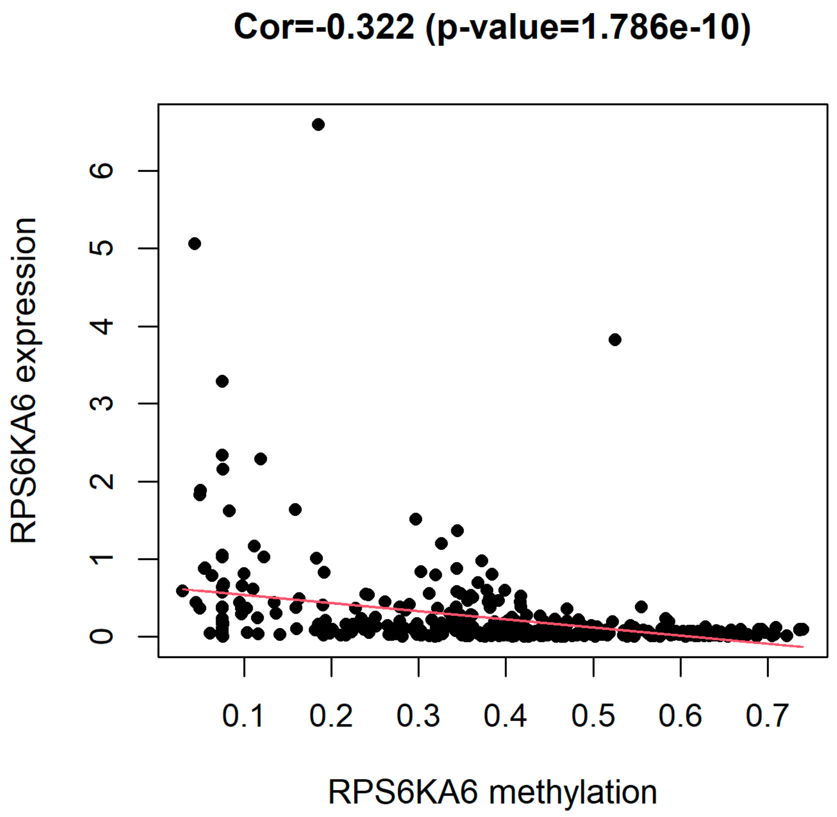
Cor=-0.322 (p-value=1.786e-10)
RPS6KA6 expression
0
1
2
3
4
5
6
0.1
0.2
0.3
0.4
0.5
0.6
0.7
RPS6KA6 methylation

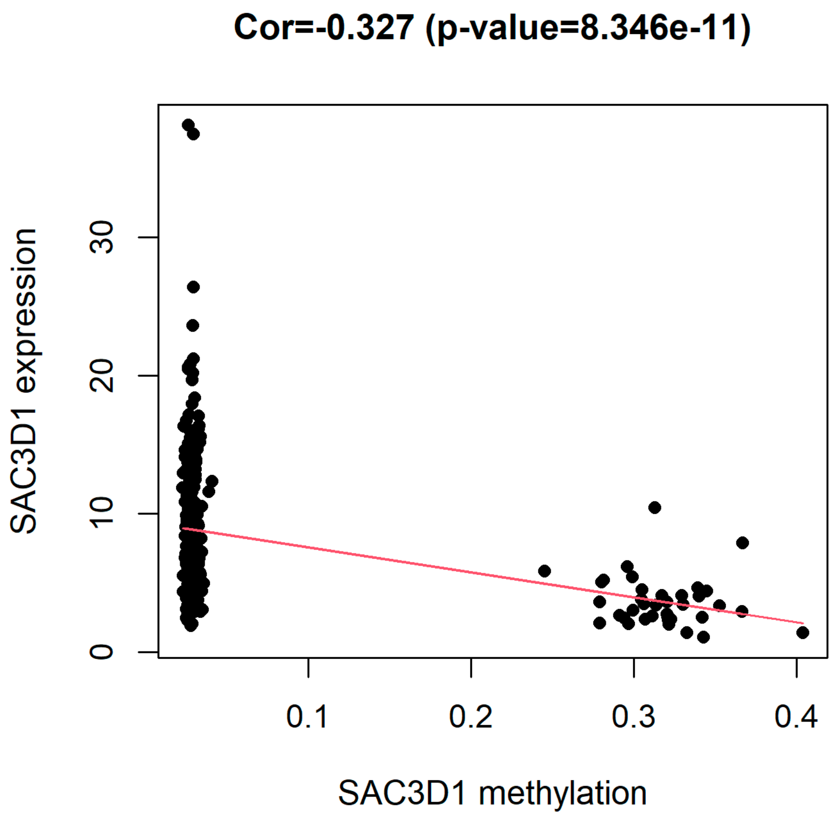
Cor=-0.327 (p-value=8.346e-11)
SAC3D1 expression
0
10
20
30
0.1
0.2
0.3
0.4
SAC3D1 methylation

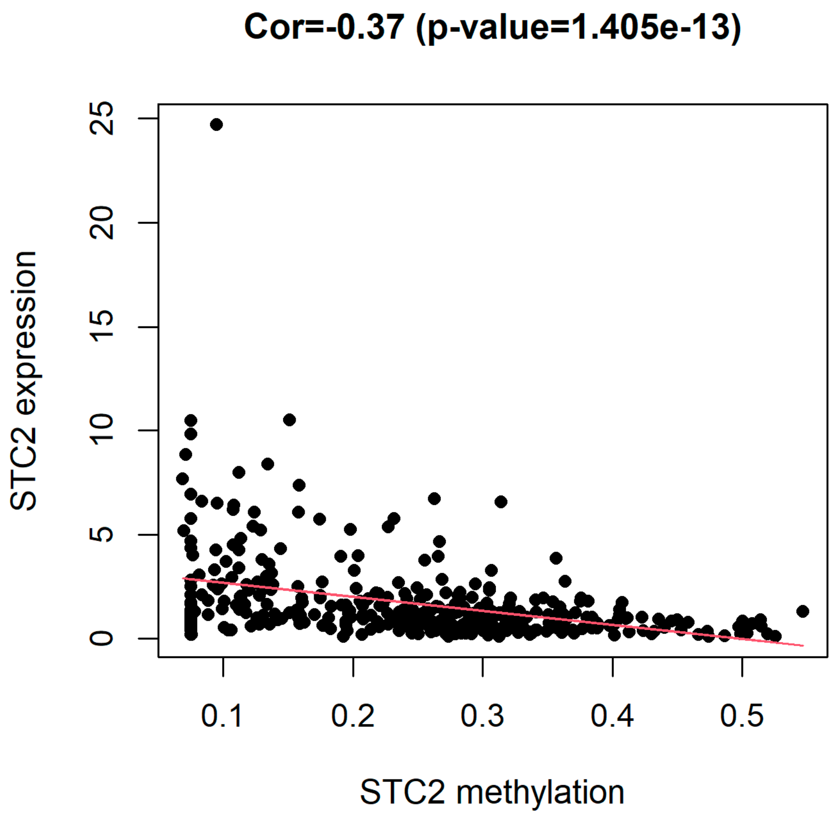
Cor=-0.37 (p-value=1.405e-13)
STC2 expression
0
5
10
15
20
25
0.1
0.2
0.3
0.4
0.5
STC2 methylation

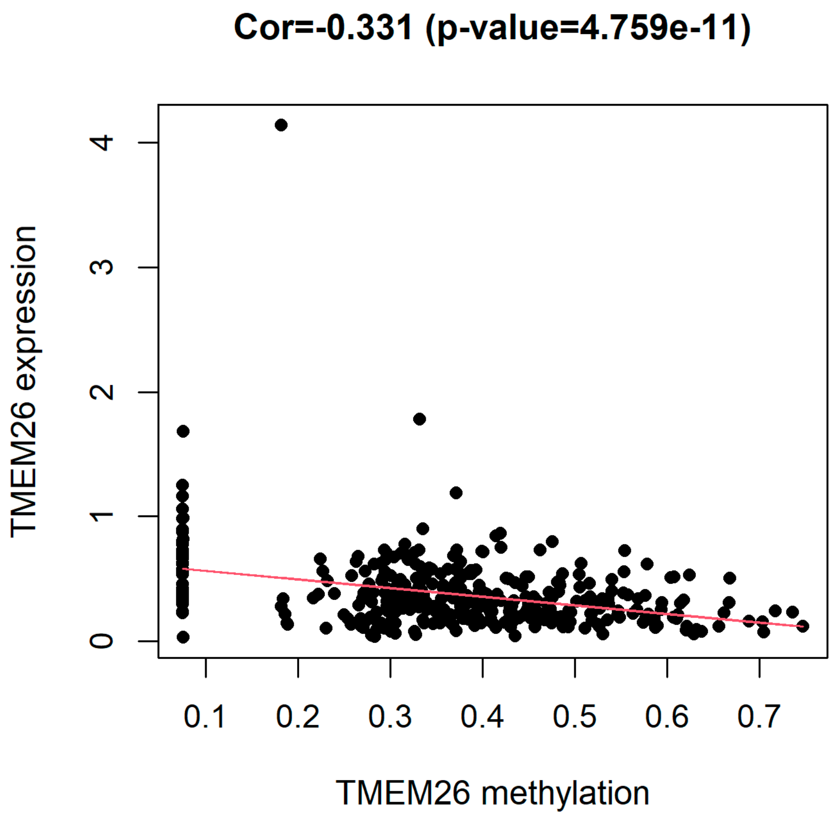
Cor=-0.331 (p-value=4.759e-11)
TMEM26 expression
0
1
2
3
4
0.1
0.2
0.3
0.4
0.5
0.6
0.7
TMEM26 methylation

Cor=-0.318 (p-value=2.861e-10)

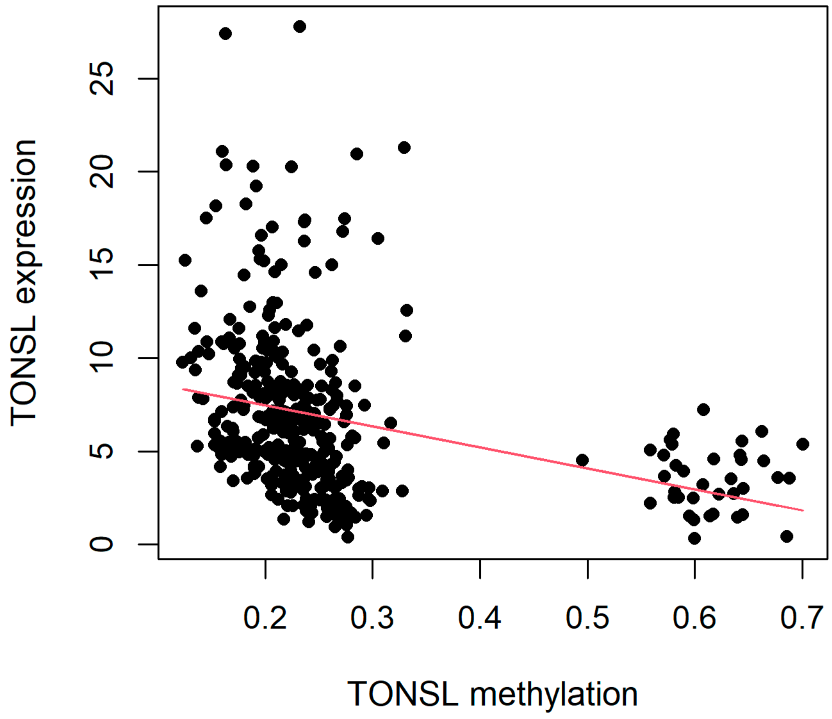

Cor=-0.385 (p-value=1.06e-14)

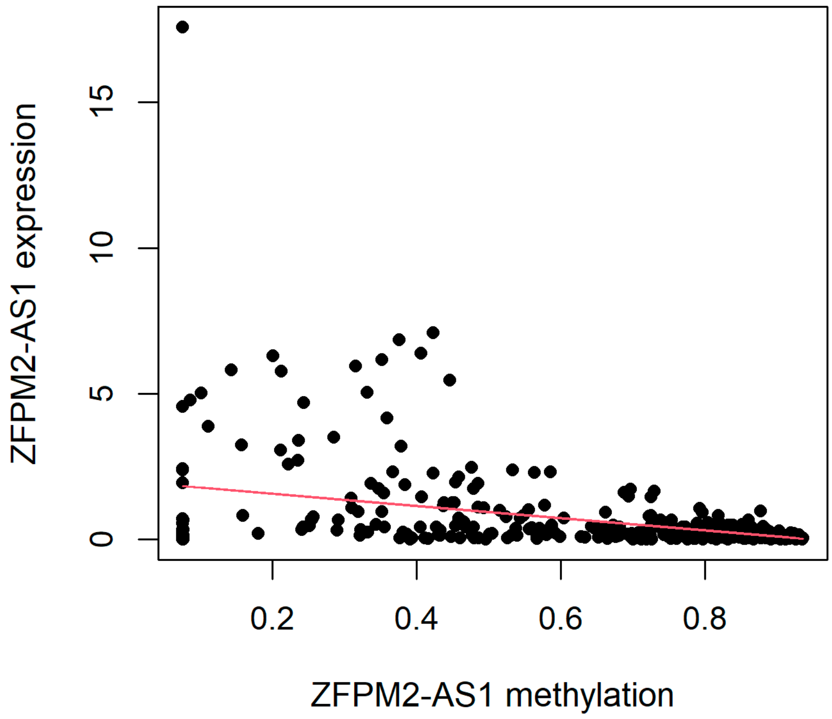

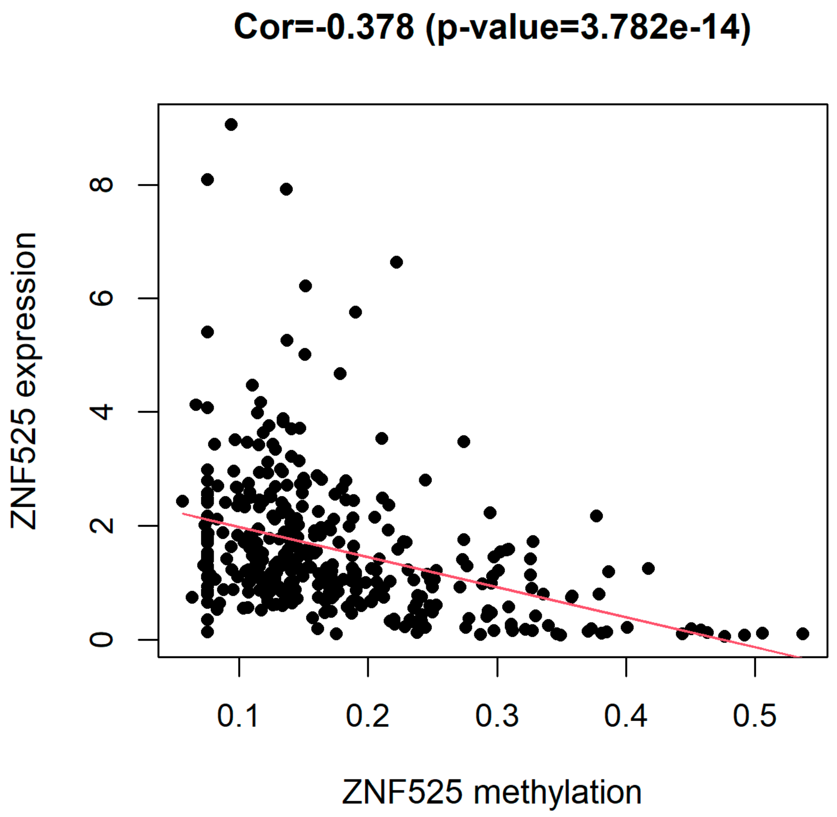
Cor=-0.378 (p-value=3.782e-14)
ZNF525 expression
0
2
4
6
8
0.1
0.2
0.3
0.4
0.5
ZNF525 methylation

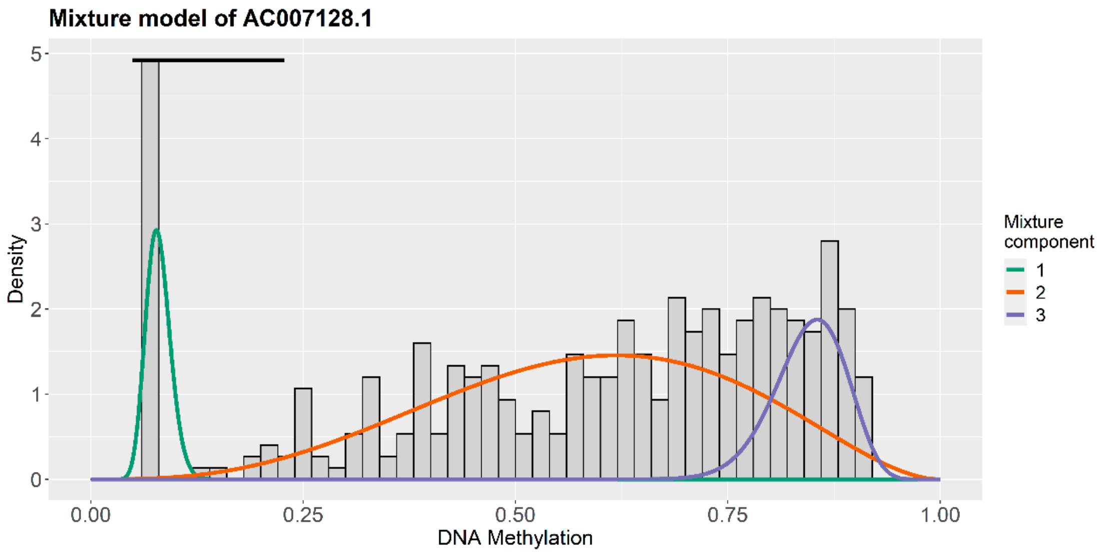
Mixture model of AC007128.1
Density
0
1
2
3
4
5
0.00
0.25
0.50
0.75
1.00
DNA Methylation
Mixture component
1
2
3

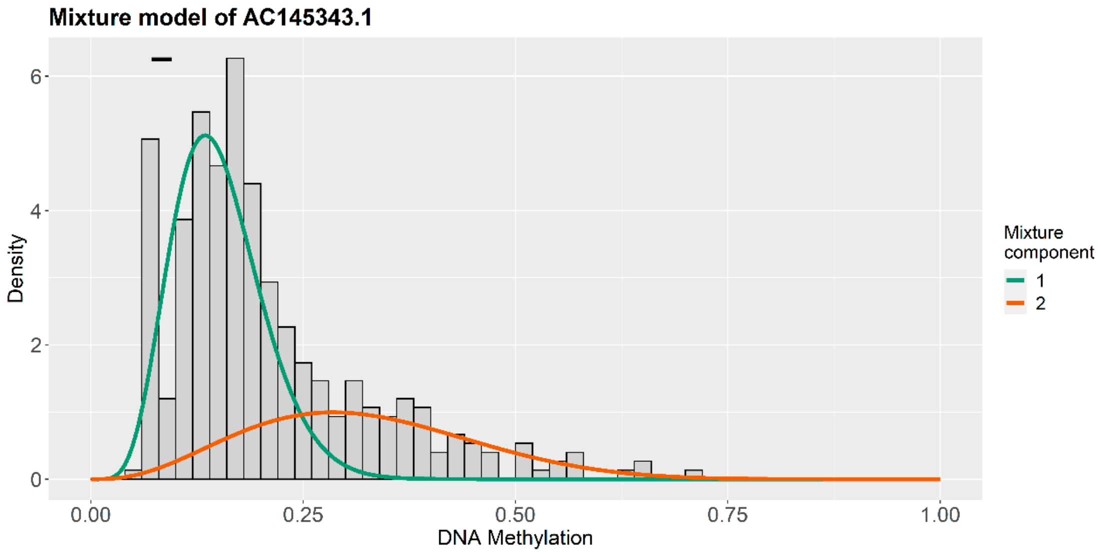
Mixture model of AC145343.1
Density
DNA Methylation
0.00
0.25
0.50
0.75
1.00
0
2
4
6
Mixture component
1
2

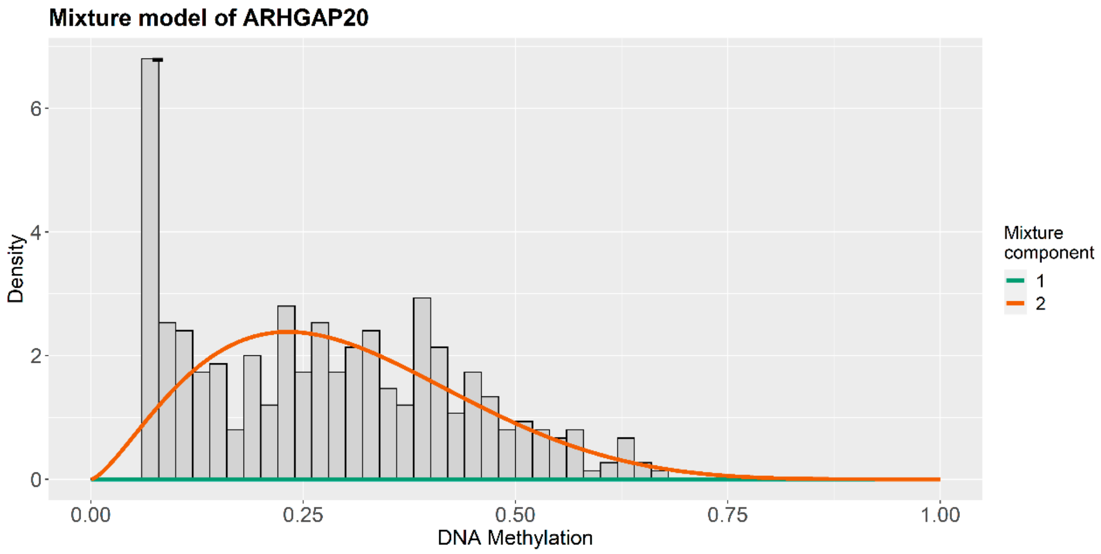
Mixture model of ARHGAP20
Density
DNA Methylation
0.00
0.25
0.50
0.75
1.00
0
2
4
6
Mixture component
1
2

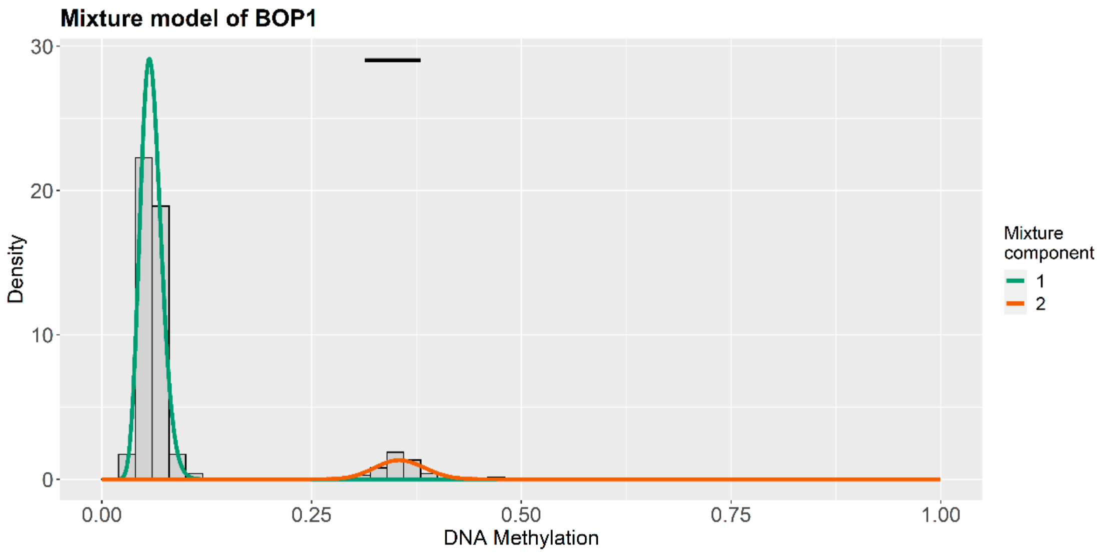
Mixture model of BOP1
Density
DNA Methylation
0.00
0.25
0.50
0.75
1.00
0
10
20
30
Mixture component
1
2

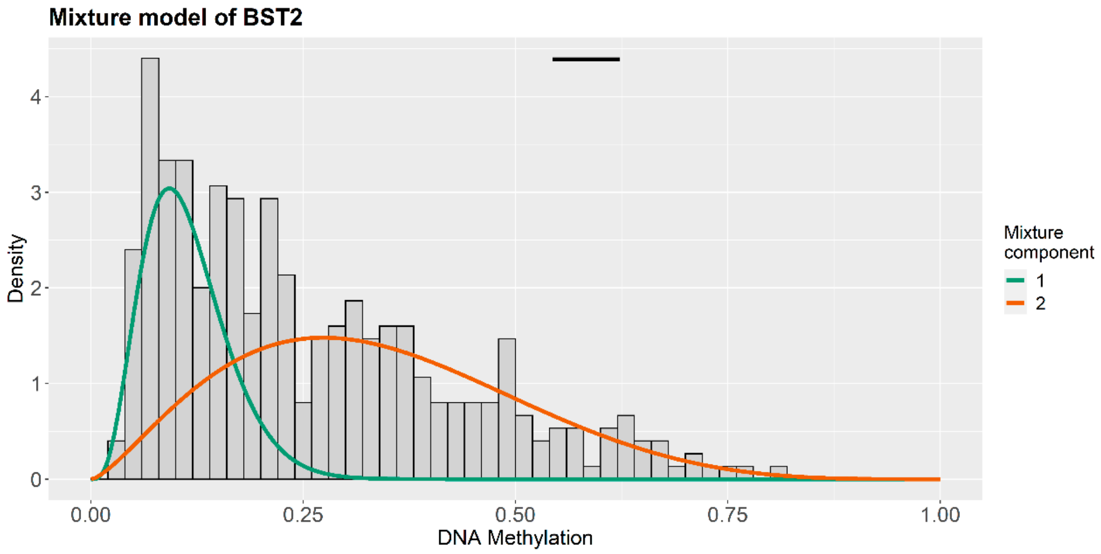
Mixture model of BST2
Density
DNA Methylation
0.00
0.25
0.50
0.75
1.00
0
1
2
3
4
Mixture component
1
2

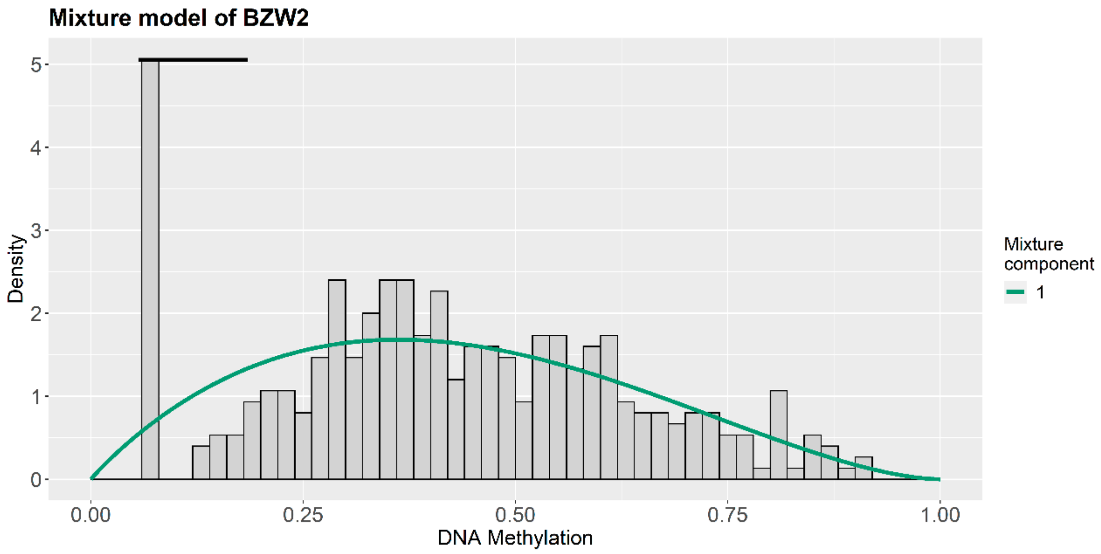
Mixture model of BZW2
Density
DNA Methylation
0.00
0.25
0.50
0.75
1.00
0
1
2
3
4
5
Mixture component
1

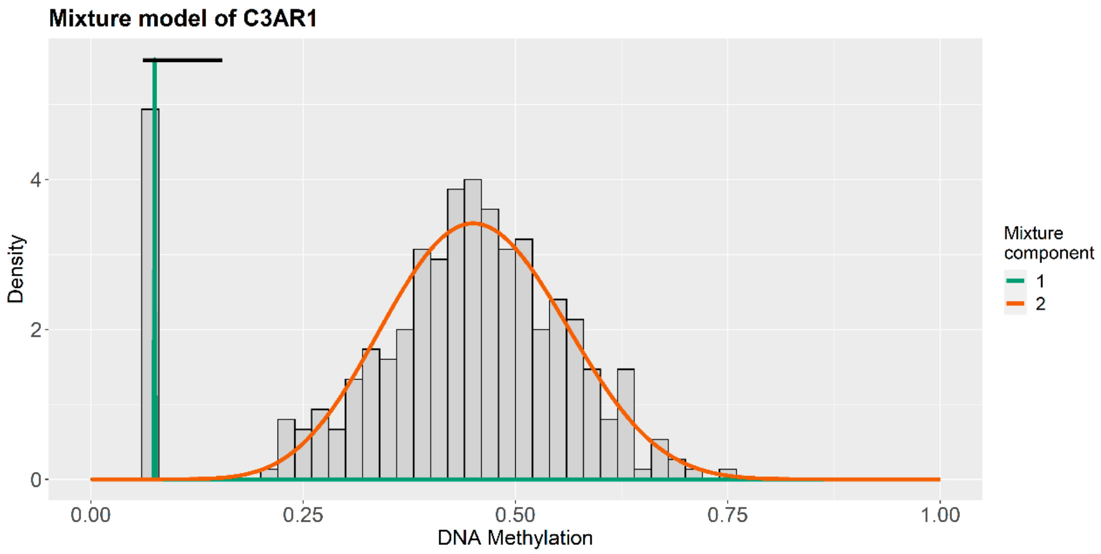
Mixture model of C3AR1
Density
DNA Methylation
0.00
0.25
0.50
0.75
1.00
0
2
4
Mixture component
1
2

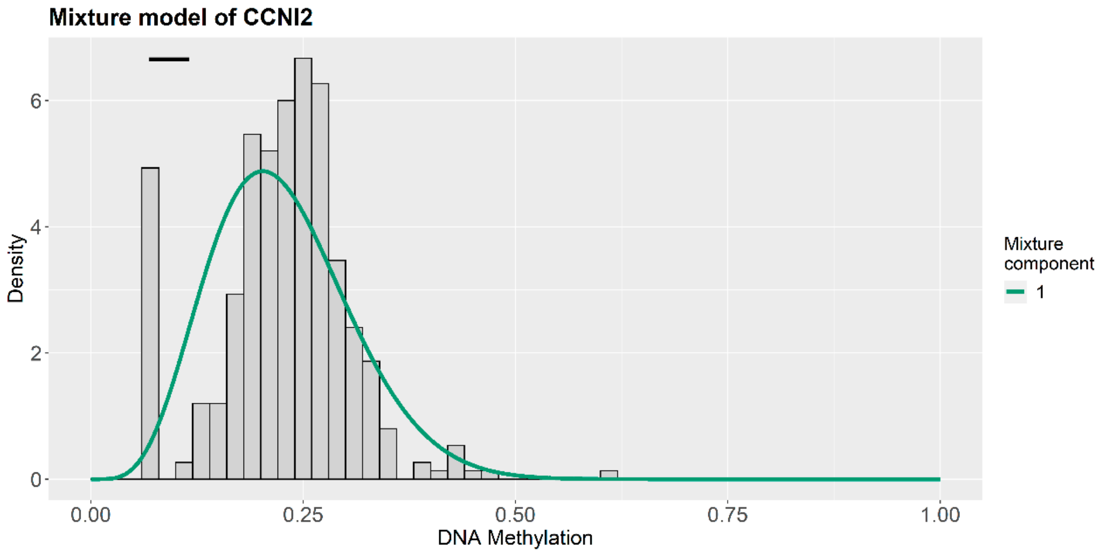
Mixture model of CCNI2
Density
0
2
4
6
0.00
0.25
0.50
0.75
1.00
DNA Methylation
Mixture
component
1

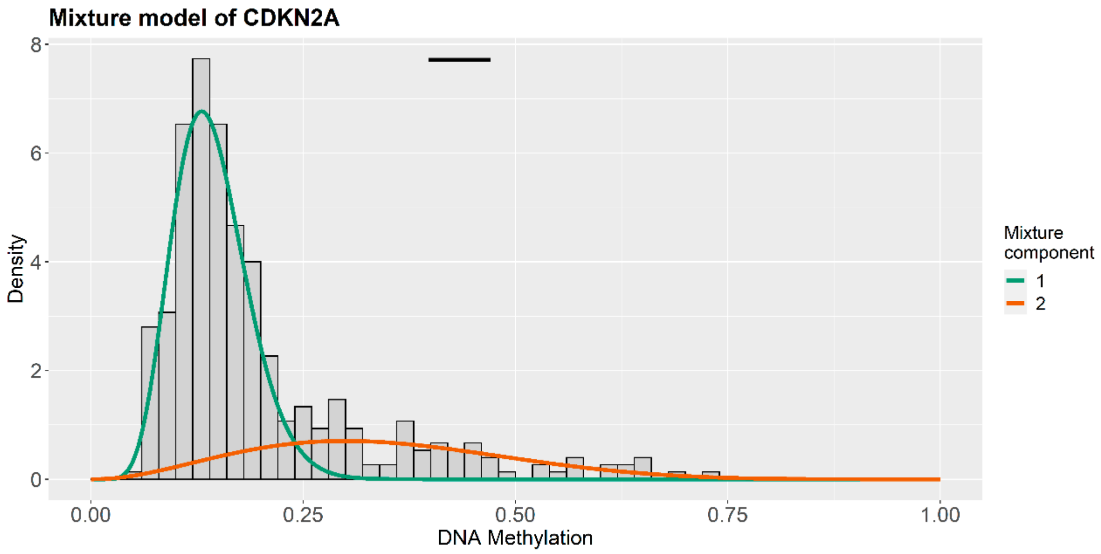
Mixture model of CDKN2A
Density
0
2
4
6
8
0.00
0.25
0.50
0.75
1.00
DNA Methylation
Mixture
component
1
2

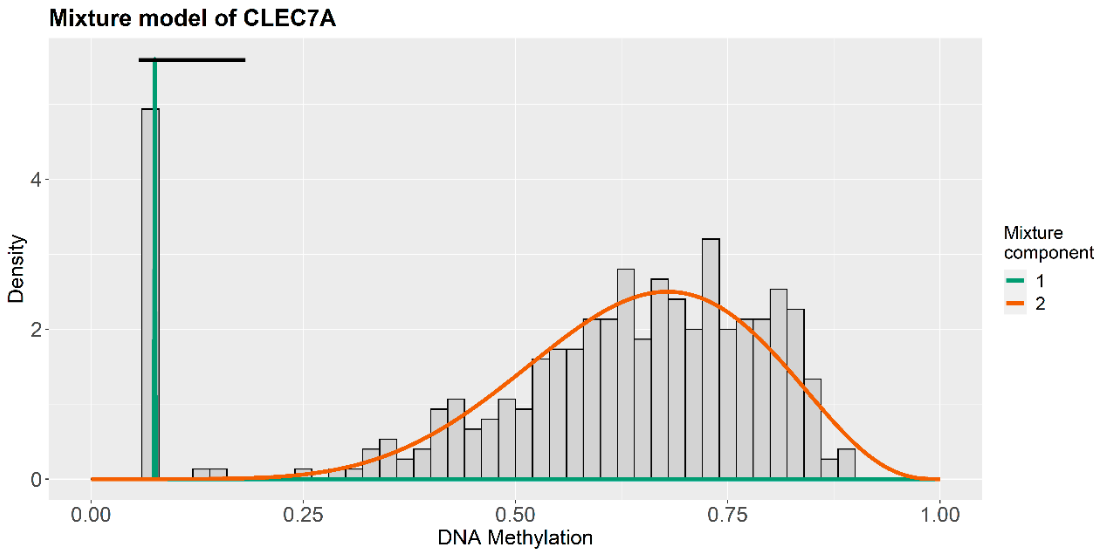
Mixture model of CLEC7A
Density
0
2
4
0.00
0.25
0.50
0.75
1.00
DNA Methylation
Mixture
component
1
2

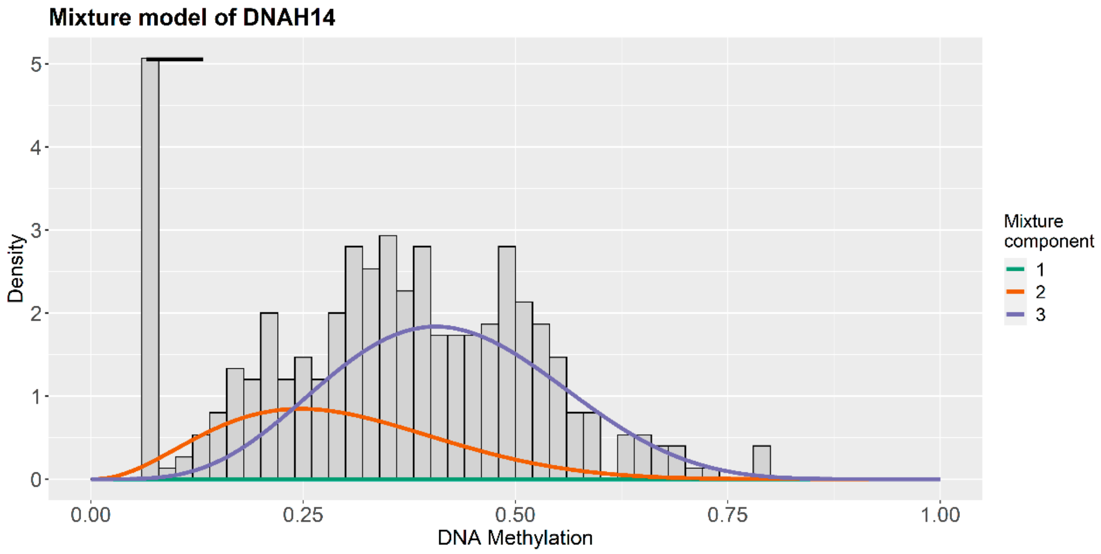
Mixture model of DNAH14
Density
DNA Methylation
0.00
0.25
0.50
0.75
1.00
0
1
2
3
4
5
Mixture component
1
2
3

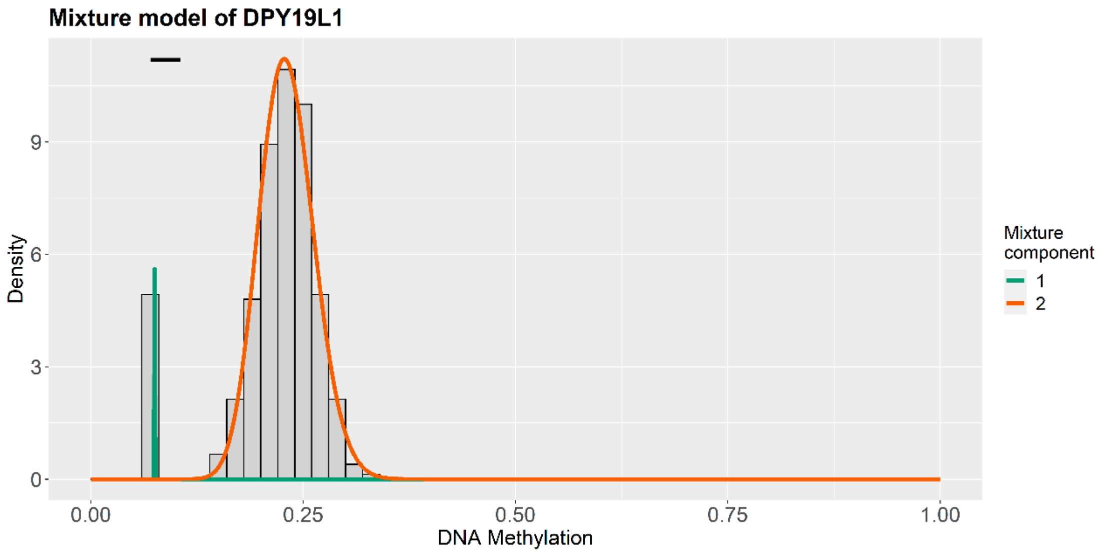
Mixture model of DPY19L1
Density
DNA Methylation
0.00
0.25
0.50
0.75
1.00
0
3
6
9
Mixture component
1
2

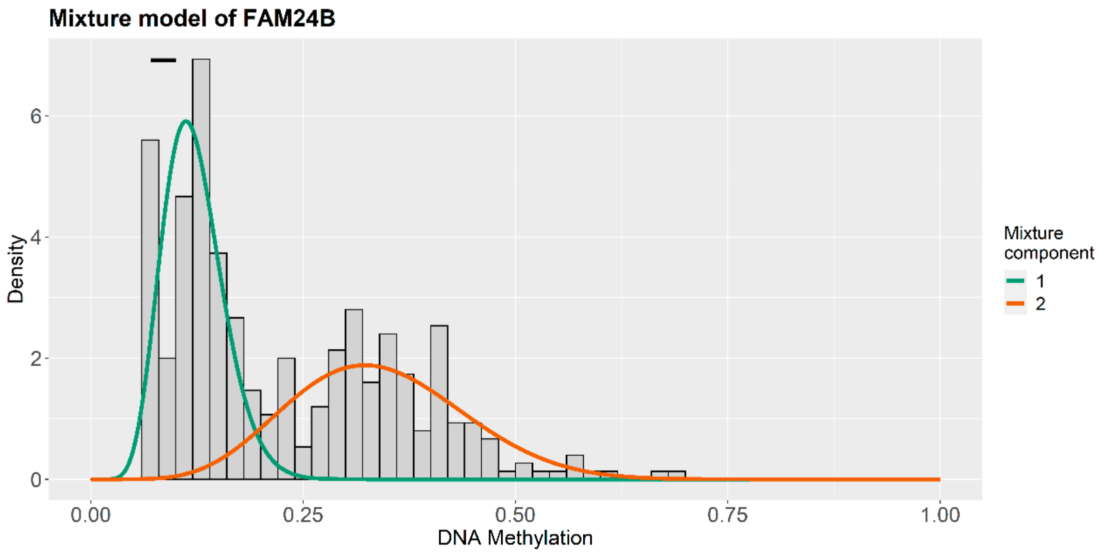
Mixture model of FAM24B
Density
DNA Methylation
0.00
0.25
0.50
0.75
1.00
0
2
4
6
Mixture component
1
2

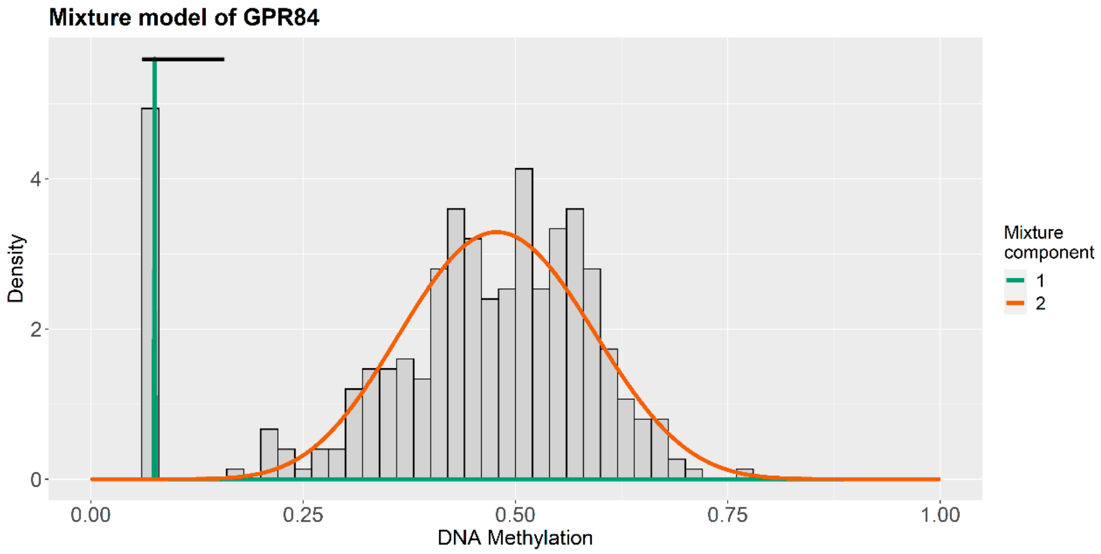
Mixture model of GPR84
Density
DNA Methylation
0.00
0.25
0.50
0.75
1.00
0
2
4
Mixture component
1
2

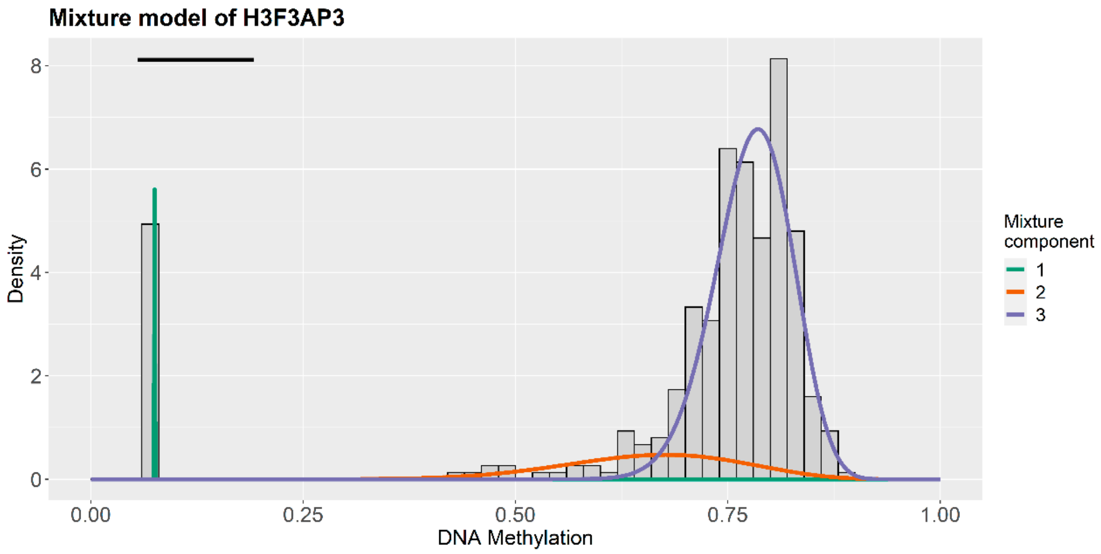
Mixture model of H3F3AP3
Density
DNA Methylation
0.00
0.25
0.50
0.75
1.00
0
2
4
6
8
Mixture component
1
2
3

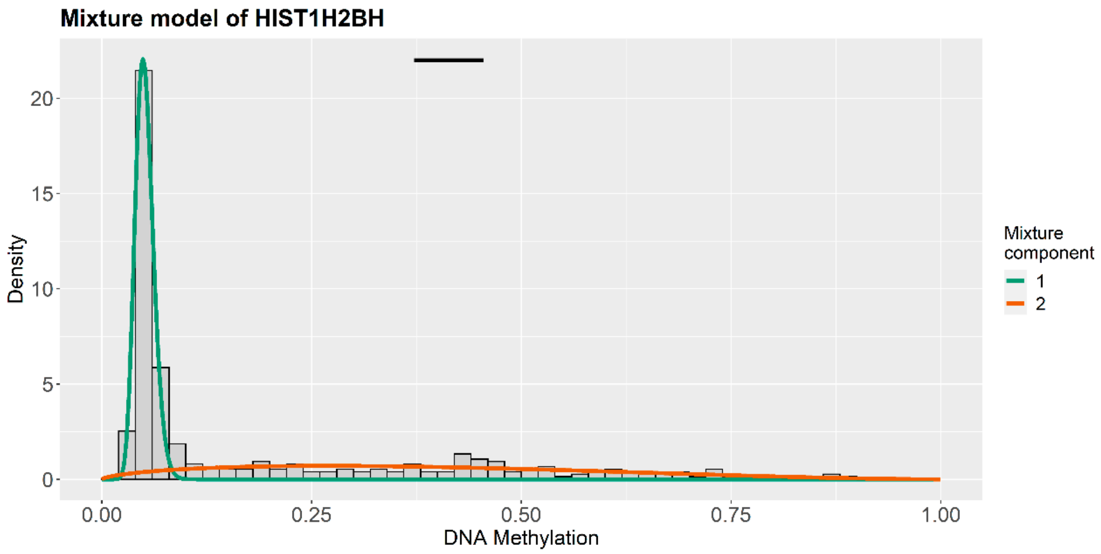
Mixture model of HIST1H2BH
Density
DNA Methylation
0.00
0.25
0.50
0.75
1.00
0
5
10
15
20
Mixture component
1
2

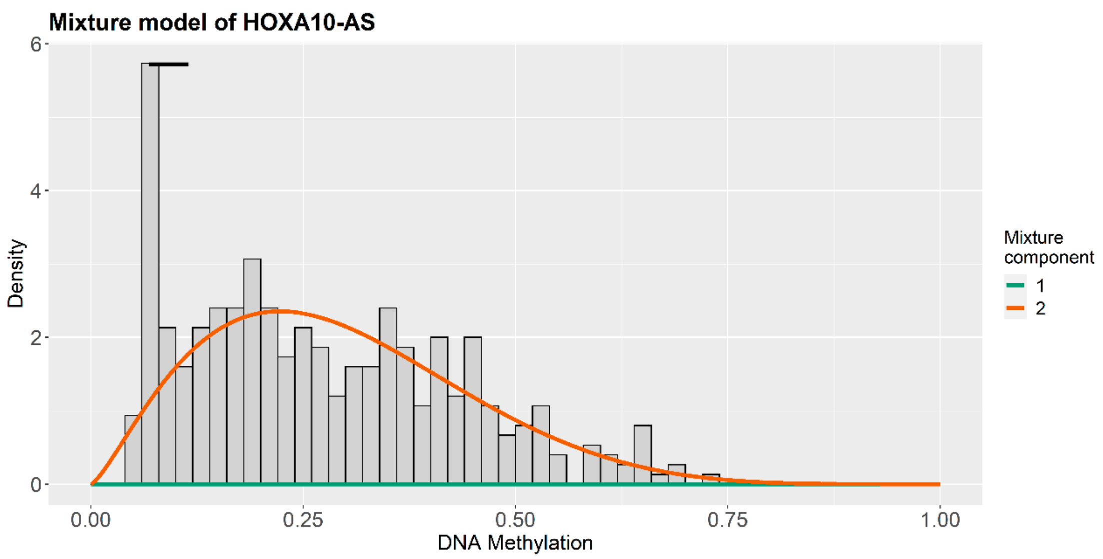
Mixture model of HOXA10-AS
Density
DNA Methylation
0
2
4
6
0.00
0.25
0.50
0.75
1.00
Mixture component
1
2

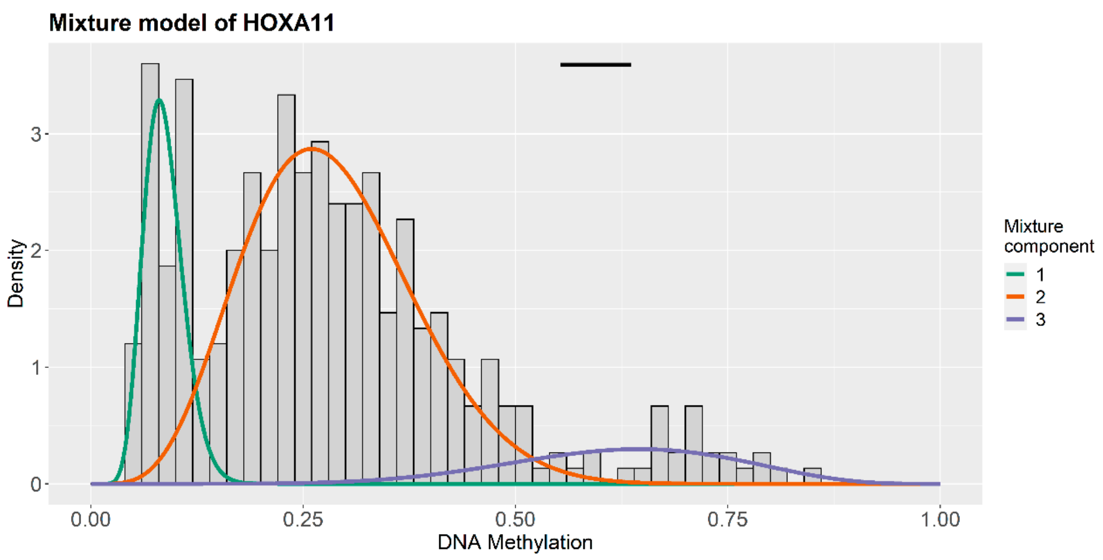
Mixture model of HOXA11
Density
DNA Methylation
0
1
2
3
0.00
0.25
0.50
0.75
1.00
Mixture component
1
2
3

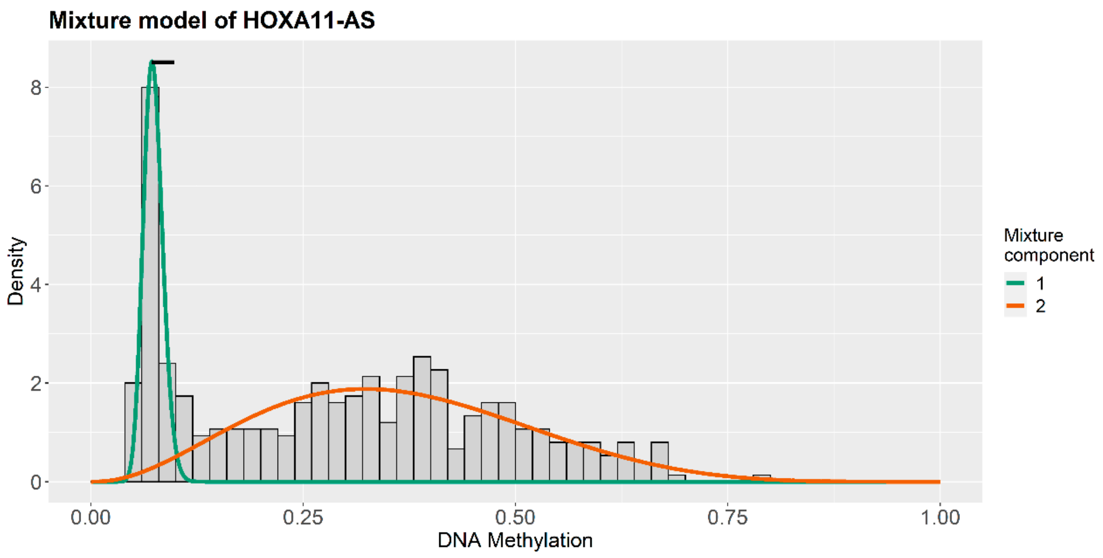
Mixture model of HOXA11-AS
Density
DNA Methylation
0
2
4
6
8
0.00
0.25
0.50
0.75
1.00
Mixture component
1
2

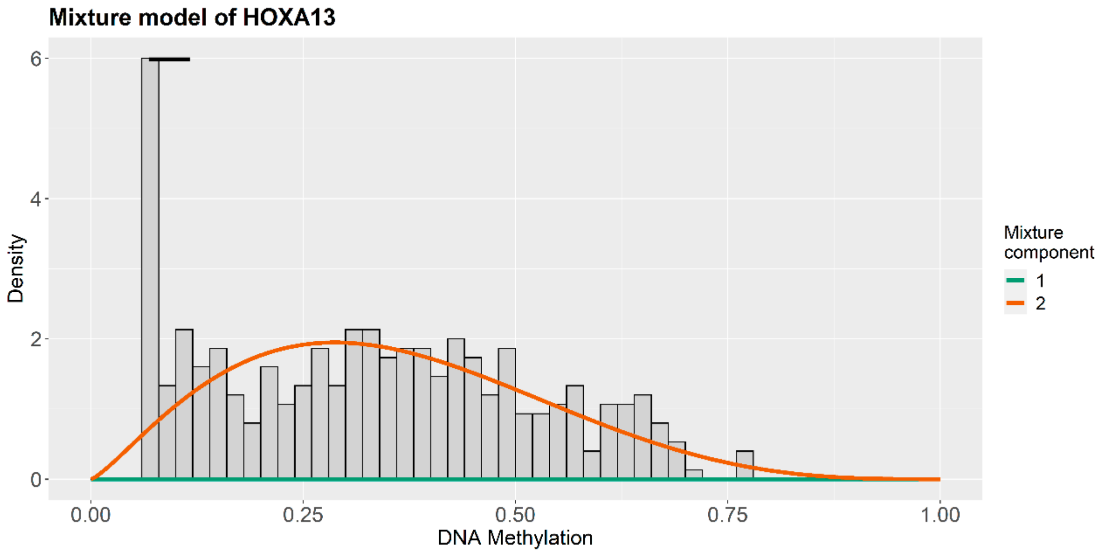
Mixture model of HOXA13
Density
DNA Methylation
0
2
4
6
0.00
0.25
0.50
0.75
1.00
Mixture component
1
2

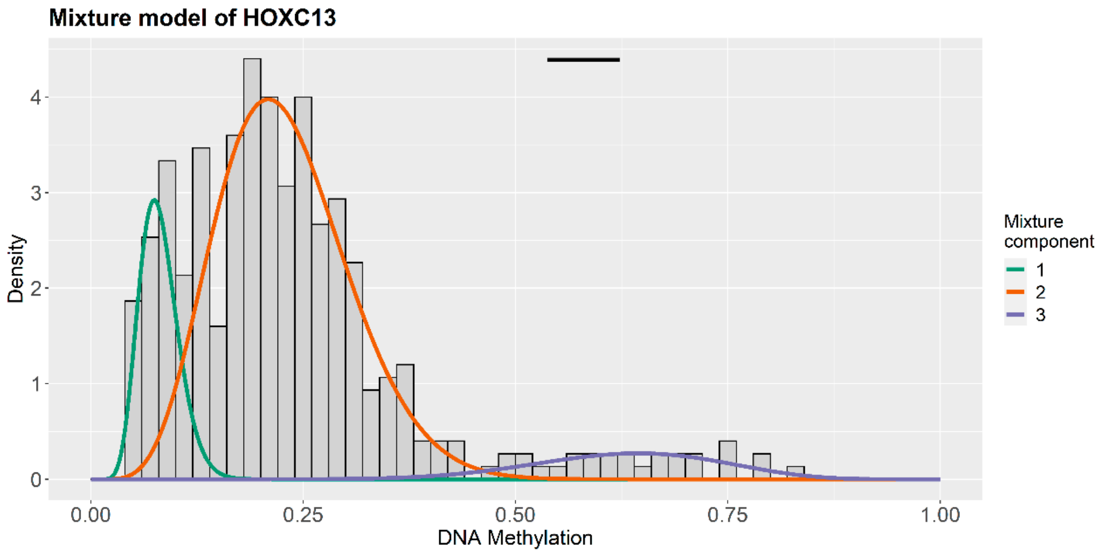
Mixture model of HOXC13
Density
DNA Methylation
0
1
2
3
4
0.00
0.25
0.50
0.75
1.00
Mixture component
1
2
3

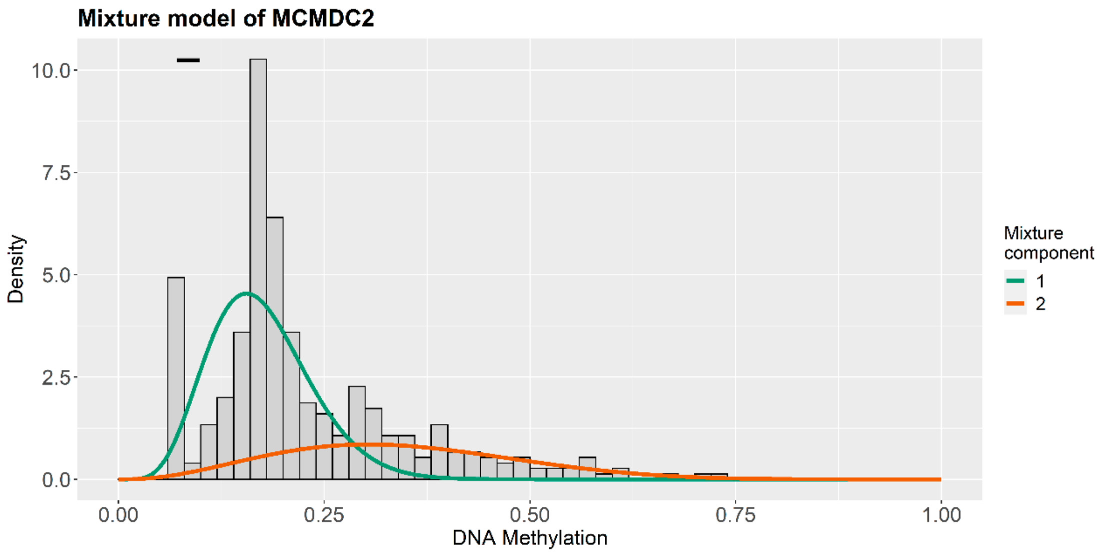
Mixture model of MCMDC2
Density
DNA Methylation
0.0
2.5
5.0
7.5
10.0
0.00
0.25
0.50
0.75
1.00
Mixture component
1
2

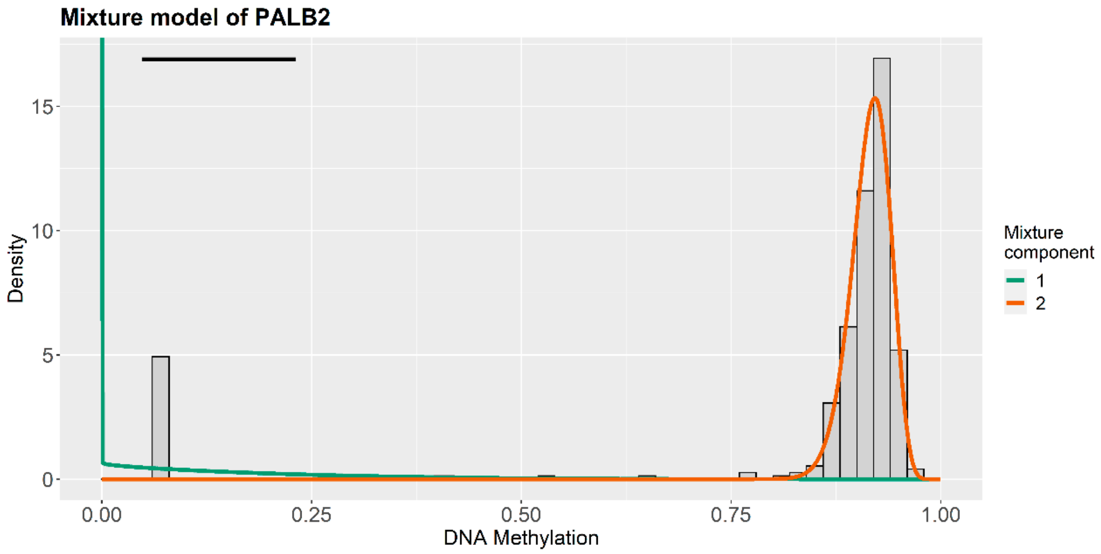
Mixture model of PALB2
Density
15
10
5
0
0.00
0.25
0.50
0.75
1.00
DNA Methylation
Mixture component
1
2

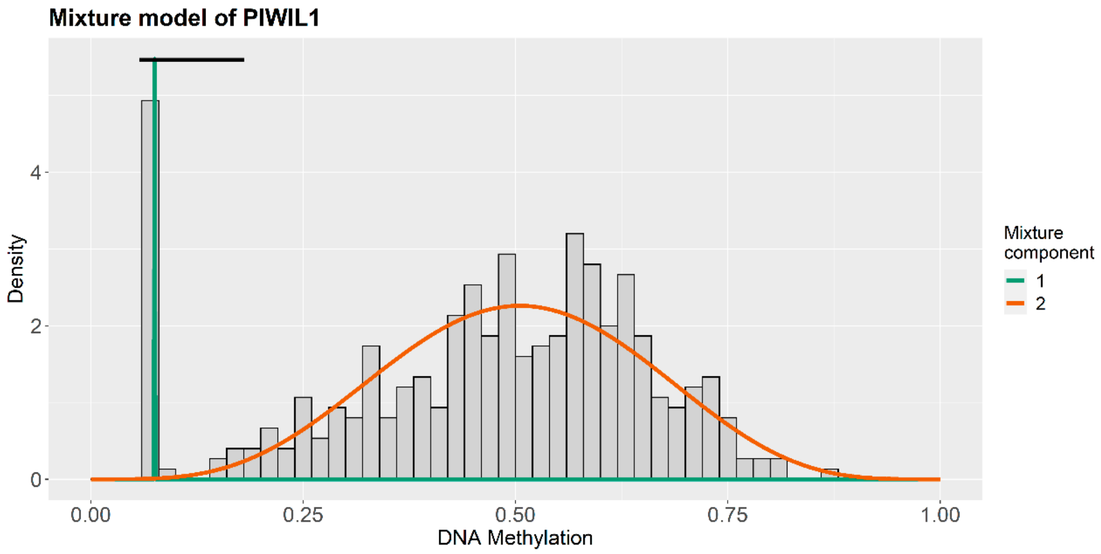
Mixture model of PIWIL1
Density
4
2
0
0.00
0.25
0.50
0.75
1.00
DNA Methylation
Mixture component
1
2

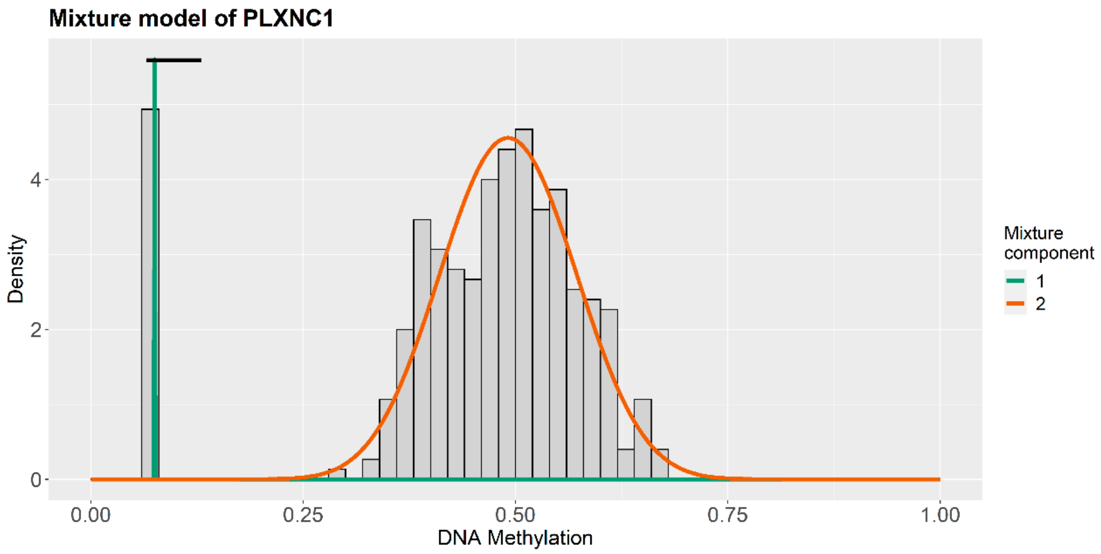
Mixture model of PLXNC1
Density
4
2
0
0.00
0.25
0.50
0.75
1.00
DNA Methylation
Mixture component
1
2

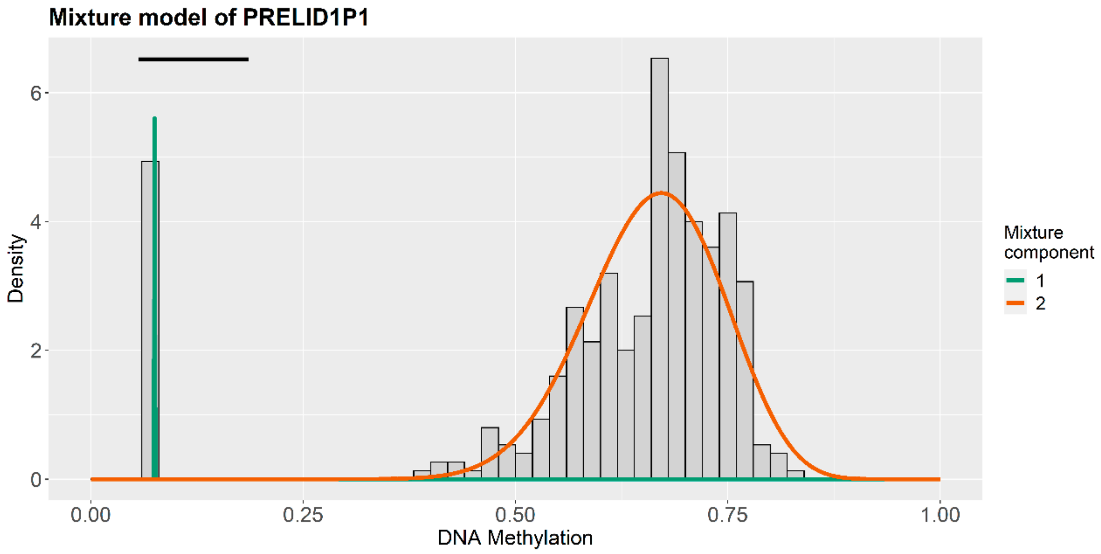
Mixture model of PRELID1P1
Density
0
2
4
6
0.00
0.25
0.50
0.75
1.00
DNA Methylation
Mixture component
1
2

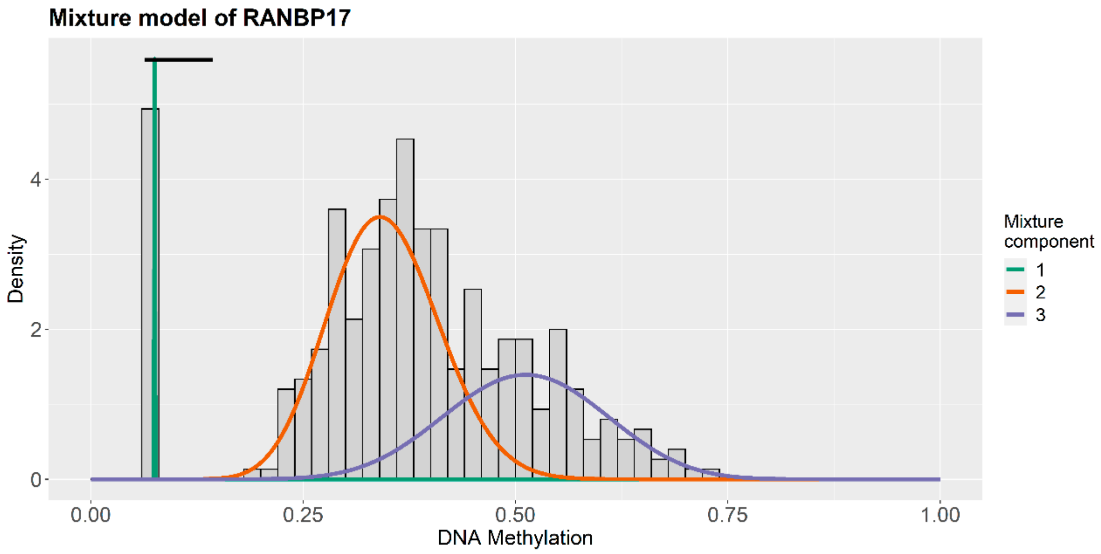
Mixture model of RANBP17
Density
0
2
4
0.00
0.25
0.50
0.75
1.00
DNA Methylation
Mixture component
1
2
3

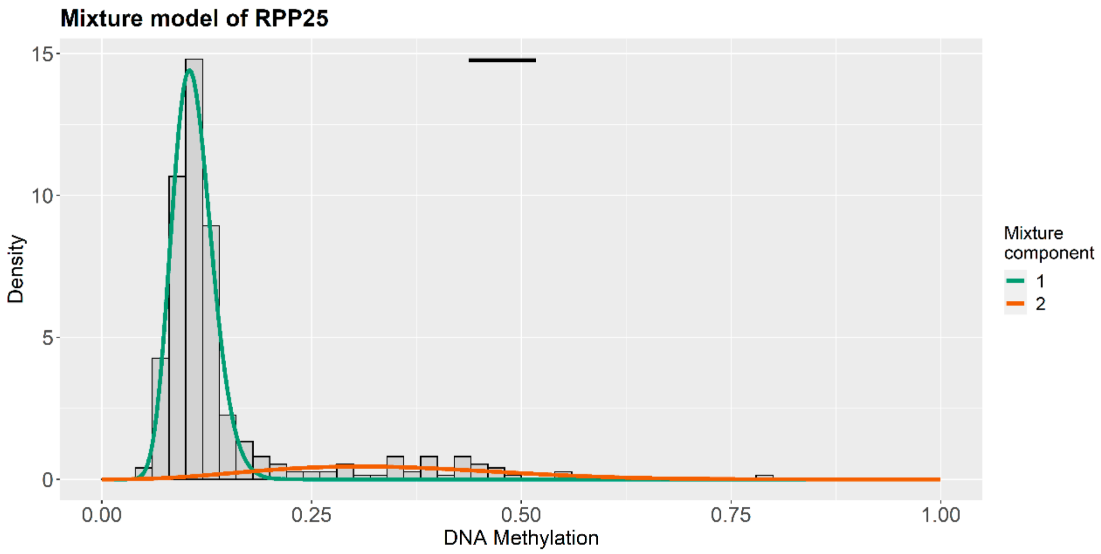
Mixture model of RPP25
Density
0
5
10
15
0.00
0.25
0.50
0.75
1.00
DNA Methylation
Mixture component
1
2

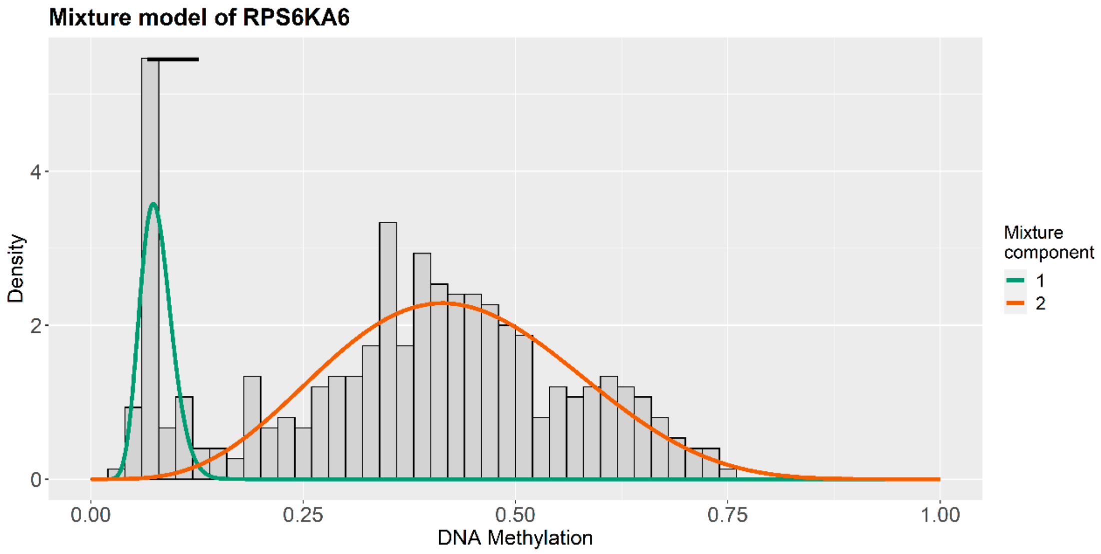
Mixture model of RPS6KA6
Density
0
2
4
0.00
0.25
0.50
0.75
1.00
DNA Methylation
Mixture component
1
2

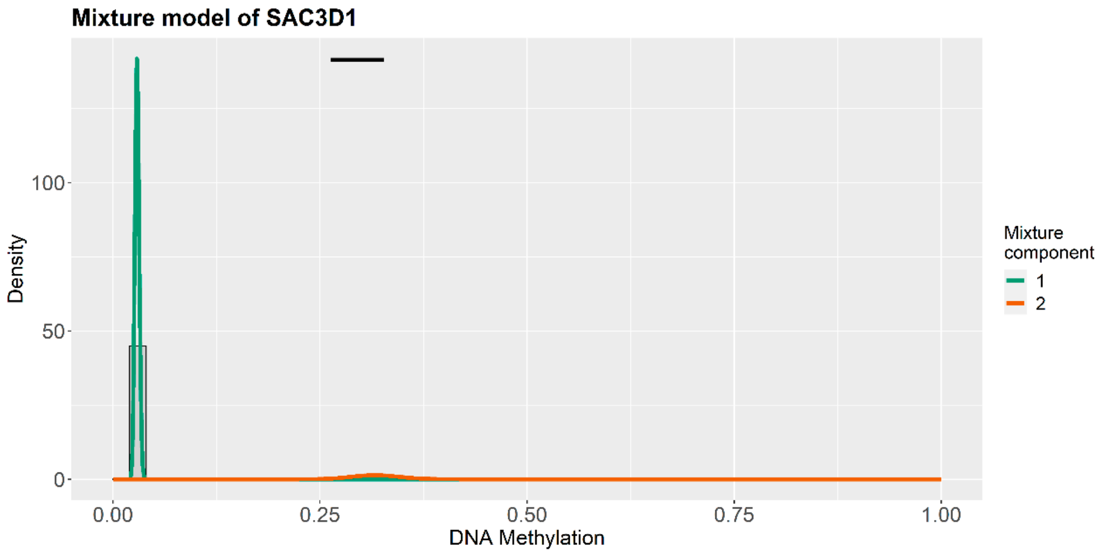
Mixture model of SAC3D1
Density
0
50
100
0.00
0.25
0.50
0.75
1.00
DNA Methylation
Mixture component
1
2

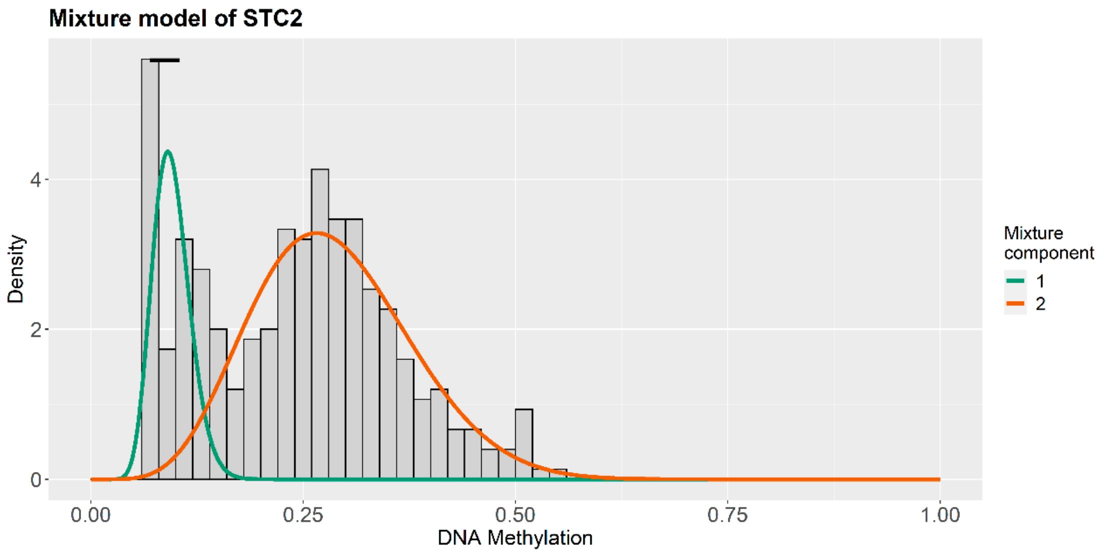
Mixture model of STC2
Density
0
2
4
0.00
0.25
0.50
0.75
1.00
DNA Methylation
Mixture component
1
2

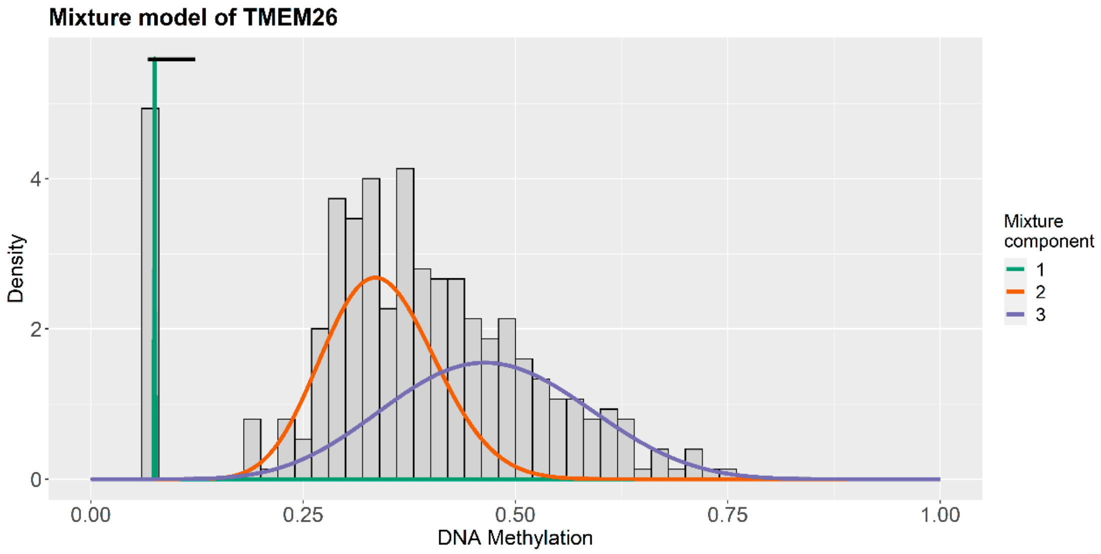
Mixture model of TMEM26
Density
0
2
4
0.00
0.25
0.50
0.75
1.00
DNA Methylation
Mixture component
1
2
3

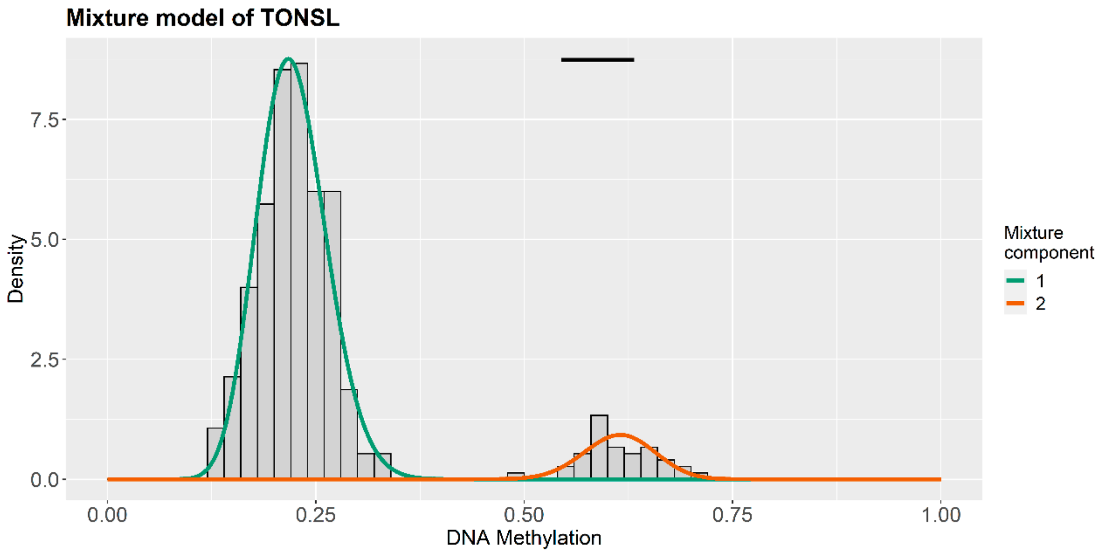
Mixture model of TONSL
Density
0.0
2.5
5.0
7.5
0.00
0.25
0.50
0.75
1.00
DNA Methylation
Mixture component
1
2

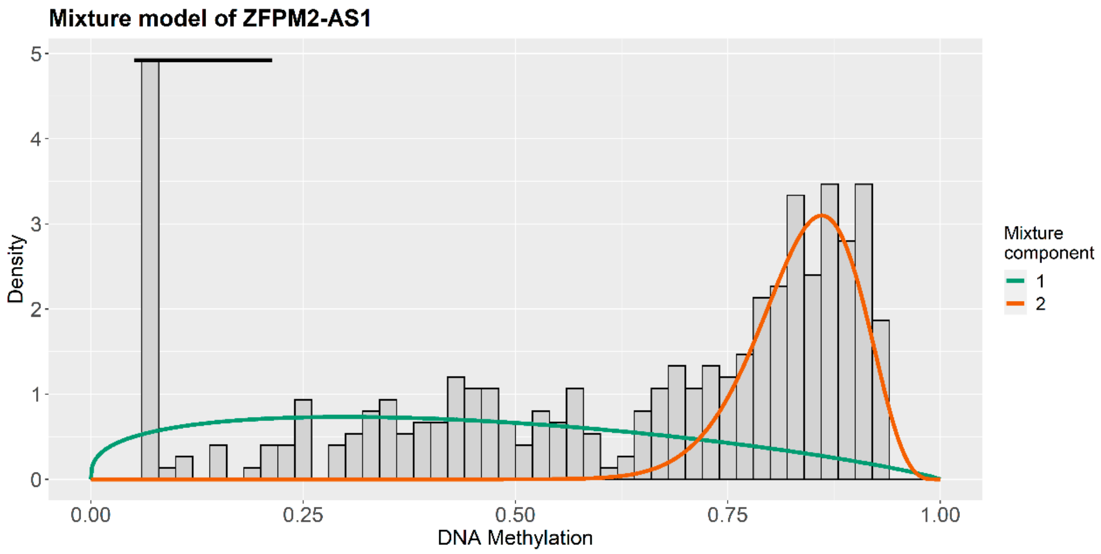
Mixture model of ZFPM2-AS1
Density
0
1
2
3
4
5
0.00
0.25
0.50
0.75
1.00
DNA Methylation
Mixture component
1
2

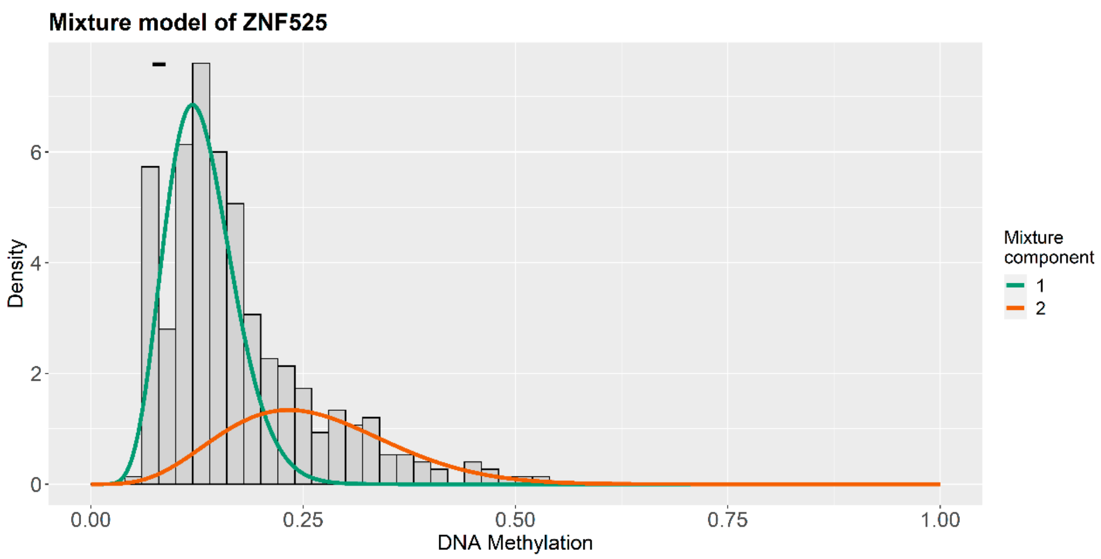
Mixture model of ZNF525
Density
DNA Methylation
0
2
4
6
0.00
0.25
0.50
0.75
1.00
Mixture component
1
2
